# Insights into the phylogenetic diversity, biological activities, and biosynthetic potential of mangrove rhizosphere Actinobacteria from Hainan Island

**DOI:** 10.3389/fmicb.2023.1157601

**Published:** 2023-05-31

**Authors:** Jing-jing Ye, Ren-jian Zou, Dong-dong Zhou, Xiao-lin Deng, Ni-lin Wu, Dan-dan Chen, Jing Xu

**Affiliations:** Collaborative Innovation Center of Ecological Civilization, School of Chemical Engineering and Technology, Hainan University, Haikou, China

**Keywords:** mangrove rhizosphere soil, phylogenetic diversity, biological activities screening, biosynthetic potential, Actinobacteria

## Abstract

Mangrove rhizosphere soils host diverse Actinobacteria tolerant to numerous stresses and are inevitably capable of exhibiting excellent biological activity by producing impressive numbers of bioactive natural products, including those with potential medicinal applications. In this study, we applied an integrated strategy of combining phylogenetic diversity, biological activities, and biosynthetic gene clusters (BGCs) screening approach to investigate the biotechnological importance of Actinobacteria isolated from mangrove rhizosphere soils from Hainan Island. The actinobacterial isolates were identifified using a combination of colony morphological characteristics and 16S rRNA gene sequence analysis. Based on the results of PCR-detected BGCs screening, type I and II polyketide synthase (PKS) and non-ribosomal synthetase (NRPS) genes were detected. Crude extracts of 87 representative isolates were subjected to antimicrobial evaluation by determining the minimum inhibitory concentration of each strain against six indicator microorganisms, anticancer activities were determined on human cancer cell lines HepG2, HeLa, and HCT-116 using an MTT colorimetric assay, and immunosuppressive activities against the proliferation of Con A-induced T murine splenic lymphocytes *in vitro*. A total of 287 actinobacterial isolates affiliated to 10 genera in eight families of six orders were isolated from five different mangrove rhizosphere soil samples, specififically, *Streptomyces* (68.29%) and *Micromonospora* (16.03%), of which 87 representative strains were selected for phylogenetic analysis. The crude extracts of 39 isolates (44.83%) showed antimicrobial activity against at least one of the six tested indicator pathogens, especially ethyl acetate extracts of A-30 (*Streptomyces parvulus*), which could inhibit the growth of six microbes with MIC values reaching 7.8 μg/mL against *Staphylococcus aureus* and its resistant strain, compared to the clinical antibiotic ciproflfloxacin. Furthermore, 79 crude extracts (90.80%) and 48 (55.17%) of the isolates displayed anticancer and immunosuppressive activities, respectively. Besides, four rare strains exhibited potent immunosuppressive activity against the proliferation of Con A-induced T murine splenic lymphocyte *in vitro* with an inhibition rate over 60% at 10 μg/mL. Type I and II polyketide synthase (PKS) and non-ribosomal synthetase (NRPS) genes were detected in 49.43, 66.67, and 88.51% of the 87 Actinobacteria, respectively. Signifificantly, these strains (26 isolates, 29.89%) harbored PKS I, PKS II, and NRPS genes in their genomes. Nevertheless, their bioactivity is independent of BGCs in this study. Our findings highlighted the antimicrobial, immunosuppressive, and anticancer potential of mangrove rhizosphere Actinobacteria from Hainan Island and the biosynthetic prospects of exploiting the corresponding bioactive natural product.

## Introduction

Natural products have served as powerful therapeutics against emerging infectious diseases and multidrug-resistant human pathogens since the golden age of antibiotics of the mid-twentieth century (Rossiter et al., 2017). However, their indiscriminate use has led to a relentless and pernicious emergence of antibiotic-resistant bacteria, along with the rapid development of cross-resistance with antibiotics worldwide (Zhou et al., 2022). They account for ~700,000 deaths annually and have already become a global challenge to public health (Antimicrobial, 2022). Hence, new antimicrobial agents are urgently needed in the pipelines of the pharmaceutical industry. Cancer incidence and mortality have always been a concern to the community, and it has been recorded as the second leading cause of death worldwide, following cardiovascular disease (Are et al., 2019). Indeed, over 60% of clinically approved anticancer agents in the past 40 years have been natural compound-derived (Sung et al., 2021). Immunosuppressants are imperative for organ recipients and patients suffering from immune-associated diseases, such as rheumatoid arthritis, membranous nephropathy, systemic lupus erythematosus, and autoimmune encephalomyelitis (Xu et al., 2021; Zhang et al., 2021b). However, most of the immunosuppressive drugs clinically used, including the most effective immunosuppressants cyclosporin A (CsA) and tacrolimus (FK506), have been reported to possess severe side effects, including nephrotoxicity and neurotoxicity. Therefore, developing new immunosuppressive drugs is highly desirable (Cen et al., 2013). Approximately 500,000 natural compounds have been reported worldwide thus far from biological sources, of which ~70,000 are microbially-derived compounds (Bérdy, 2012). Of the 23,000 known bioactive secondary metabolites, 10,100 (45%) are reported to be produced by Actinobacteria (Xu et al., 2014).

Actinobacteria are gram-positive microorganisms with high genomic G+C rations in their DNA. It represents one of the valid publications of the names of 42 phyla of prokaryotes, which are ubiquitous in nature (Hong et al., 2009; Salam et al., 2020; Oren and Garrity, 2021, 2022). Members of Actinobacteria phylum, especially the genus *Streptomyces*, are demonstrated to have the most significant advantage as natural metabolite producers, notably antibiotics (e.g., streptomycin, erythromycin, tetracycline, chloramphenicol, vancomycin, and thienamycin), immunosuppressive agents (e.g., tacrolimus, rapamycin, and kanglemycin C), anti-tumor agents (e.g., dactinomycin, daunorubicin, doxorubicin, mitomycin, and plicamycin), and enzymes and enzyme inhibitors (Valli et al., 2012). However, the increased recognition of the difficulty of finding pharmaceutically significant metabolites through traditionally in-depth investigated terrestrial environments has become more challenging. Thus, bioprospecting studies have advanced into Actinobacteria from unusual or specialized ecological niches, such as mangroves (Law et al., 2019; Lu et al., 2021).

Mangroves are widely distributed in the transition zone between land and sea, mainly spreading along tropical and sub-tropical estuaries zones. They are famous for their unique environment with highly productive ecosystems (Li et al., 2022). In China, mangroves are usually found along the South China Sea Coast, most widely distributed on Hainan Island (Zhang et al., 2007). Its extreme environmental factors, such as high salinity, low oxygen, tidal gradients, high temperature, and excessively high intensity light, could act as an effective selector for metabolic pathway evolution of active actinobacterial community *via* the generation of structurally unprecedented and biologically interesting metabolites of pharmaceutical importance (Pavan Kumar et al., 2018; Li T. et al., 2019). Hitherto, 1,000's of Actinobacteria affiliated with 96 genera in 35 families have been isolated and identified from mangroves since 2014, mainly from the soil. According to the frequency of their appearance, *Streptomyces, Micromonospora, Nocardiopsis, Rhodococcus*, and *Microbacterium*, have been recognized as the predominant culturable mangrove Actinobacteria (Xu et al., 2022). At least 86 new species affiliated with eight novel genera have been isolated and identified from mangroves, most of which are affiliated with the genus *Streptomyces* (Li F. N. et al., 2019; Lu et al., 2021; http://www.bacterio.net/). Furthermore, ~200 compounds, including some “hot biomolecules,” such as salinosporamide A (to be processed for clinical trials for cancer treatment), antibiotic drug leads, including xiamycins, rifamycins, and antimycin A were discovered from mangrove Actinobacteria (Xu, 2011, 2015; Jiang et al., 2018; Li et al., 2022).

Encoding a wealth of biosynthetic gene clusters (BGCs) makes Actinobacteria well-known producers of diverse secondary metabolites (Kamjam et al., 2017; Matsumoto and Takahashi, 2017; Singh et al., 2021). Among these gene clusters, polyketide synthase (PKS) and non-ribosomal peptide synthetase (NRPS) genes are the most commonly used to assess the natural product potential of a community due to their involvement in the biosynthesis of numerous bioactive compounds through multifunctional pathways (Marfil-Santana et al., 2021). Polyketides (PKs) such as erythromycin and rapamycin are encoded in type I polyketide synthase (PKS-I), and tetracycline and doxorubicin are assembled using type II polyketide synthase (PKS-II) gene clusters (Seow et al., 1997; Hertweck et al., 2007; Gontang et al., 2010; Kharel et al., 2012; Rao et al., 2016). On the other hand, non-ribosomal peptides (NRPs) such as antibiotics daptomycin (Kirkpatrick et al., 2003) and vancomycin (Chau et al., 2011) are synthesized by NRPS. Thus, far, a majority of the screenings for biosynthetic potential are based on amplification of particular domains of the gene clusters using degenerated primers based on the conserved nature of PKS and NRPS modules, more specifically the PKS I ketosynthase (KS) domain, PKS II KSα domain, and NPRS adenylation (AD) domain, respectively, thereby enabling to analyze the variability of the gene cluster (Liu et al., 2016; Wei et al., 2018). However, limited efforts have been devoted to exploring actinomycetic diversity, biological activities, and biosynthetic potential for PKs and NRPs discovery associated with rhizosphere Actinobacteria of mangroves in Hainan Island.

Thus, the present study aims to investigate the biodiversity of cultivable Actinobacteria of mangrove rhizosphere soils collected from various sites of Hainan Island and assess their biosynthetic potential as producers of compounds with antimicrobial, anticancer, and immunosuppressive properties. The obtained data will offer a basis for the follow-up exploiting the corresponding bioactive natural product.

## Materials and methods

### Sample location and collection

**Figure 1:**
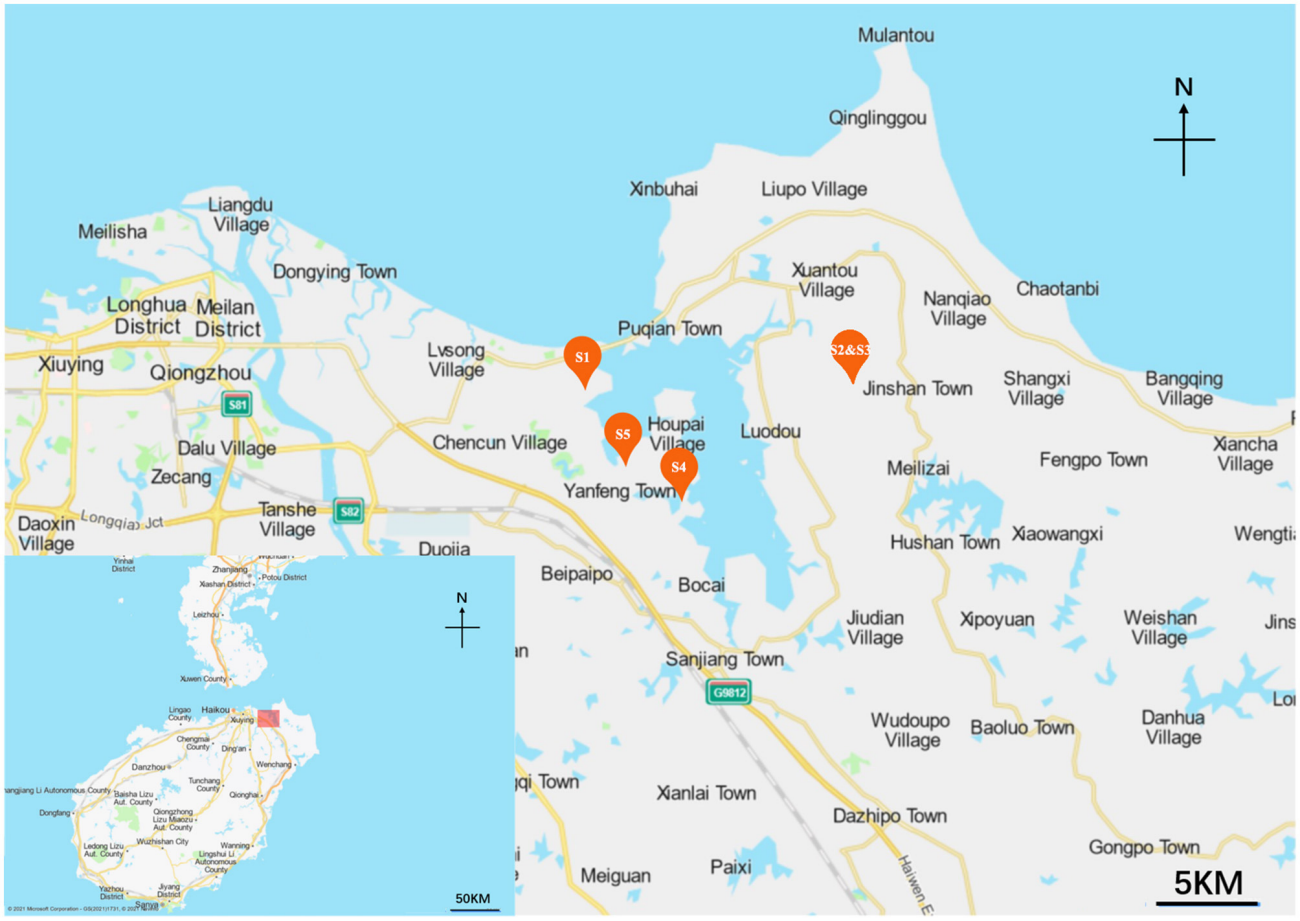
The map of the sampling sites along Hainan Island, China.

Five mangrove rhizosphere soil samples were collected in 2021 at different mangrove sites of Hainan Island, Hainan province, China. The locations where the samples were collected and their information are shown in Figure 1, Supplementary Table 1. The map was made by Baidu Map (https://api.map.baidu.com/lbsapi/createmap/), and there are no copyright issues. All samples (serially named S1, S2, S3, S4, and S5) were collected from depths 10–15 cm from the main roots. Five parallel soil samples at each site within a 20 m^2^ area were mixed to obtain one sample, then packed in sterile bags and transported to the laboratory quickly. All samples were air-dried before grinding with a mortar and pestle.

### Cultivable Actinobacteria isolation and maintenance

Seven media were prepared to isolate the actinobacterial strains (Supplementary Table 2). All media were supplemented with nalidixic acid (20 mg/L), cycloheximide (50 mg/L), and potassium dichromate (50 mg/L) to inhibit the growth of gram-negative bacteria and fungi. Strains were isolated using the dilution plating technique as described by Ye et al. (2018). Each soil sample (S1–S5; 5 g) was dissolved in 45 mL of sterile water and shaken for 3 h at 180 rpm at room temperature to release actinobacterial cells attached to the soil. The pretreated samples were subsequently prepared for serial 10-fold dilutions up to 10^−4^. An aliquot of diluted suspensions (10^−2^, 10^−3^, 10^−4^ of 0.2 mL) was spread in triplicate onto agar plates of the above media and incubated at 28°C for 1–4 weeks. From each plate, all phenotypically distinct colonies were picked and purified by repeating streaking using a sterile L-shaped loop onto freshly prepared ISP2 agar plates until pure isolates were obtained. Putative actinobacterial colonies were selected based on colony morphology, the color of aerial and substrate mycelium, the production of diffusible pigments, and the growth rate. The pure individual strains were cryopreserved on ISP2 agar slants at 4°C for short-term storage and as glycerol suspensions (20%, v/v) at −80°C for long-term storage.

### DNA extraction, PCR amplification, sequencing, and phylogenetic analysis of 16S rRNA gene

Genomic DNA was extracted from the isolated 287 strains grown for 7 days in 50 mL ISP2 medium at 28°C using the Chelex-100 method described previously by Lu et al. (2019). The DNA was applied as a PCR template for 16S rRNA gene amplification using polymerase chain reaction (PCR). The primers used for PCR were 27F (5′-AGAGTTTGATCCTGGCTCAG-3′) and 1492R (5′-GGTTACCTTGTTACGACTT-3′) (DeLong, 1992). The PCR mixtures (50 μL) contained 25 μL 2 × supermix (TransGen, Beijing), 1.5 μL each of the primers (10 μM, Sangon Biotech, Beijing), 2.0 μL DNA, and 20.0 μL ddH_2_O. The following conditions were observed: initial denaturation at 95°C for 3 min, 30 cycles of denaturation at 94°C for 1 min, annealing at 60°C for 1 min, extension at 72°C for 1 min, and final extension at 72°C for 10 min. The amplicons were then visualized by gel electrophoresis in 1% agarose gel. The products were purified and sequenced on the ABI PRISMTM 3730XL DNA Analyzer (Foster City, CA). The obtained sequences were compared with the sequences in NCBI (http://www.ncbi.nlm.nih.gov/) and EzBioCloud (https://www.ezbiocloud.net/) databases (Yoon et al., 2017) using the Basic Local Alignment Search Tool (BLAST) programs for searching the sequence homology with closely related organisms. When the top three matching BLAST hits were from the same species and were ≥98% similar to the query sequence, this species name was assigned to the selected isolate. From the samples, 87 representative isolates were first selected by 16S rRNA sequences alignment and then selected with a lower similarity rate from the aligned strain. Actinobacterial 16S rRNA sequences of 87 representative isolates were deposited in GenBank under the accession numbers (OM019210-OM019296) listed in Supplementary Table 3. Multiple alignments were made using the CLUSTAL_X tool in MEGA version 7.0 (Kumar et al., 2016). A phylogenetic tree based on the neighbor-joining method (NJ) was used to infer the evolutionary history of the actinomycetes under Kimura's two-parameter model (Kimura, 1980), and the bootstrapping was carried out using 1,000 replications. Tree visualization was done via the Interactive Tree of Life (iTOL) web service (Letunic and Bork, 2019).

### Small scale-fermentation and extracts preparation

Approximately 87 representative strains were inoculated into 100 mL ISP2 broth in 500 mL conical flasks and incubated for 7 days at 28°C with shaking (180 rpm). A total of 300 mL (3 × 100 mL) cultural broth of each strain was centrifuged at 4,200 rpm at 20°C for 20 min to separate the mycelium from the broths. The supernatants were extracted with EtOAc (1:1, v/v) three times and then diluted with H_2_O to produce an aqueous suspension that was successively extracted using EtOAc. The organic layers were dried over magnesium sulfate and evaporated to dryness to obtain an EtOAc fraction (EF). The water layer was lyophilized, and its residue (WF) was collected.

Meanwhile, the mycelia were soaked overnight in acetone and then filtered. The acetone extract was dried in vacuo to obtain mycelia fraction (MF). Currently, ethyl acetate is an efficient solvent used for extracting actinobacterial culture supernatant and is environmentally friendly. Most of the un-polar and semi-polar components were transferred from the aqueous to the organic phase, and then the extracts were easily separated from water and dried by rotary evaporation. However, it was less efficient in extracting polar substances. The mycelium was ultimately extracted using acetone due to its compatibility with un-polar, semi-polar, and polar components. All the titled extracts were dissolved in dimethyl sulfoxide (DMSO, 100 mg/ml) and stored at 4°C until use.

### Antimicrobial activity screening

Six indicator microorganisms, including gram-positive *Staphylococcus aureus* (*S. aureus*; ATCC 29213, ATCC 33591), gram-negative *Pseudomonas aeruginosa* (*P. aeruginosa*; ATCC 27853), *Escherichia coli* (*E. coli*; ATCC 25922, ATCC 35218), and pathogenic *Candida albicans* (*C. Albicans*; CCTCC 93025; Microbial Culture Collection Center of Guangdong, China, CIMCC) were used to evaluate the antimicrobial activity of the extracts from the 87 actinobacterial isolates by the serial dilution technique (Zhang et al., 2021a). *S. aureus* ATCC 33591 is resistant to cefoxitin and oxacillin, and *E. coli* ATCC 35218 is resistant to ampicillin. Briefly, in each well of a 96-well microtiter plate, 2 μl of the extract was added to 198 μl of a suspension of each indicator strain. The plate was incubated for 24/48 h at 37/28°C. The minimum inhibitory concentration (MIC) was assigned to the lowest concentration that completely inhibited the growth of a given indicator microorganism. Ciprofloxacin (1 mg/mL) and amphotericin B (1 mg/mL) were used as positive controls.

### Anticancer activity screening

The anticancer screening was performed using the 3-(4,5-dimethylthiazol-2-yl)-2,5-diphenyltetrazolium bromide (MTT) method according to our previous report (Zhou et al., 2022). The human cancer cell lines, including HepG2 (liver cancer cell line), HeLa (cervical cancer cell line), and HCT-116 (colorectal cancer cell line) purchased from the Type Culture Collection of the Chinese Academy of Sciences, Shanghai, China. The cells were cultured in 96-well plates of 5,000 cells/well and maintained in RPMI-1640 culture medium with 200 μL/mL fetal bovine serum (FBS), 100 mg/ml streptomycin, and 100 U/ml penicillin under a humidified atmosphere of 5% CO_2_ and 95% air at 37°C for 24 h. Subsequently, the cells were treated with 10 μg/mL crude extracts added to each well and incubated for 24 h under the above conditions. Any traces of the extracts were removed by washing with PBS and applying medium with 20 μL MTT dissolved in 100 μL medium for 4 h at room temperature. DMSO (200 μL) was added to dissolve the formazan crystals. The absorbance was measured at 570 nm by a microplate reader. Doxorubicin (10 μg/mL) was used as the positive control.

### Immunosuppressive activity screening

Tetrazolium salt-based CCK-8 assay was used to quantify the immunosuppressive activities against the proliferation of Con A (concanavalin A)-induced T lymphocyte proliferation with some modification (Xu et al., 2021). Splenocytes collected from BALB/c female mice (6–8 weeks old, Hainan Medical University) were plated at a density of 5 × 10^6^ cells/mL onto 96-well plates containing 100 μL of RPMI 1640 complete medium. Thereafter, 50 μL concanavalin A (Con A, 20 μg/ml, Sigma) and 50 μL RPMI 1640 medium containing 40 μg/mL crude extracts were added to a final volume of 200 μL. The control group was treated with RPMI 1640 medium containing CsA or only 0.1% DMSO. After 48 h of incubation at 37°C in 5% CO_2_, 20 μL CCK-8 reagent was added to each well and then placed in the incubator to culture for 4 h in a dark condition. The absorbance of each solution was measured at 450 nm with a microplate reader. Cyclosporine A (10 μg/mL) was used as the positive control.

### PCR screening for biosynthetic genes

Based on the analysis of phenotypic and phylogenetic characteristics, 87 representative strains were selected for the screening of secondary metabolite biosynthetic genes. Three sets of degenerate primers targeting the conserved regions of biosynthetic genes were used for PCR amplification. (i) K1F (5′-TSA AGT CSA ACA TCG GBC A-3′) and M6R (5′-CGC AGG TTS CSG TAC CAG TA-3′; Metsä-Ketelä et al., 1999; Ayuso-Sacido and Genilloud, 2005; Gontang et al., 2010) targeting PKS-I ketosynthase (KS) domain using the following conditions: initial denaturation of 95°C for 5 min; 35 cycles of 95°C for 30 s, 55°C for 2 min, and 72°C for 4 min; and a final extension at 72°C for 5 min. (ii) KSαF-(5′-TSGRCTACRTCAACGCSCACGG-3′) and KSβR-(5′ -TACSAGTCSWTCGCCTGGTTC-3′; Metsä-Ketelä et al., 1999; Gontang et al., 2010) targeting PKS type II KSα domain using the following conditions: 95°C for 2 min; 35 cycles of 95°C for 1 min, 55°C for 2 min, and 72°C for 1.5 min; and a final extension at 72°C for 8.5 min. (iii) A3F (5′ -GCS TAC SYS ATS TAC ACS TCS GG-3′) and A7R (5′-SAS GTC VCC SGT SCG GTA S-3′; Metsä-Ketelä et al., 1999; Ayuso-Sacido and Genilloud, 2005; Gontang et al., 2010) targeting NRPS adenylation (AD) domain sequences using the following conditions: initial denaturation of 95°C for 5 min; 35 cycles of 95°C for 30 s, 59°C for 2 min, and 72°C for 4 min; and a final extension at 72°C for 10 min (Metsä-Ketelä et al., 1999; Gontang et al., 2010). The amplicons (corresponding to 1,200–1,400 bp for the KS domain of PKS-I, 600 bp for the KSα domain of PKS-II, and 700–800 bp for the AD domain of NRPS genes) were then visualized and purified as stated above.

### Statistical analysis

Bar plot, heat map, and α-diversity (including Richness, Shannon–Weine Diversity index (*H*′), Simpson's Diversity Index (1-*D*), Pielou, Invsimpson, and Chao1 indices) were created or calculated using R software (version 3.7.0) with Ggplot2, pPheatmap, and Vegan packages, respectively. The Bray-Curtis matrix was employed to evaluate the β-diversity and visualized using Principal Coordinate Analysis (PCoA). Results were expressed as mean ± standard deviation (SD) of triplicate measurements for the antimicrobial, immunosuppressive, and anticancer activities. The relationship of biological activities and shared isolates were analyzed using the UpSetR package in R software for antimicrobial, while ordinary one-way ANOVA (*p* < 0.05) followed by Dunnett's *t*-test using Statistical Analysis System (SPSS 13.0 software, SPSS, USA) and GraphPad Prime 7 for immunosuppressive and anticancer activities. The correlation of BGCs and representative isolates was investigated using the Venn Diagram package in R software.

## Results

### Isolation and phylogenetic analysis of culturable actinobacterial strains

To isolate Actinobacteria, mangrove rhizosphere soil samples were collected from Hainan Island in five different geographic areas (Supplementary Table 1) using serial dilution protocols and seven kinds of isolation media (Supplementary Table 2). After 1–4 weeks, individual colonies were picked from the plates based on the morphological characteristics of leathery colonies, aerial hyphae or substrate mycelia, and pigmentation ranging from white, brownish white, khaki yellow, orange, red, brown to maroon (Supplementary Figure 1). To further ascertain the taxonomic status of the isolates, a partial 16S rRNA gene was PCR-amplified and sequenced. Among 723 isolates obtained, 287 isolates were identified as belonging to the Actinobacteria class by comparing with their best match retrieved from NBCI and EzBioCloud. The BLAST analysis indicated that most isolated Actinobacteria (276 strains) displayed diverse taxonomic affinities with 98.65–100% similarity. Another 11 strains (strain No. A-57, A-58, A-62, A-83, A-85, A-112, A-208, A-325, A-499, A-591, and A-615) exhibited low 16S rRNA gene sequence identity <98.65%, the threshold for differentiating two different species (Kim et al., 2014), which indicated that these isolates could represent new taxonomic species and assigned to genus *Streptomyces* (Supplementary Table 3).

### Phylogenetic analysis and distribution

**Figure 2:**
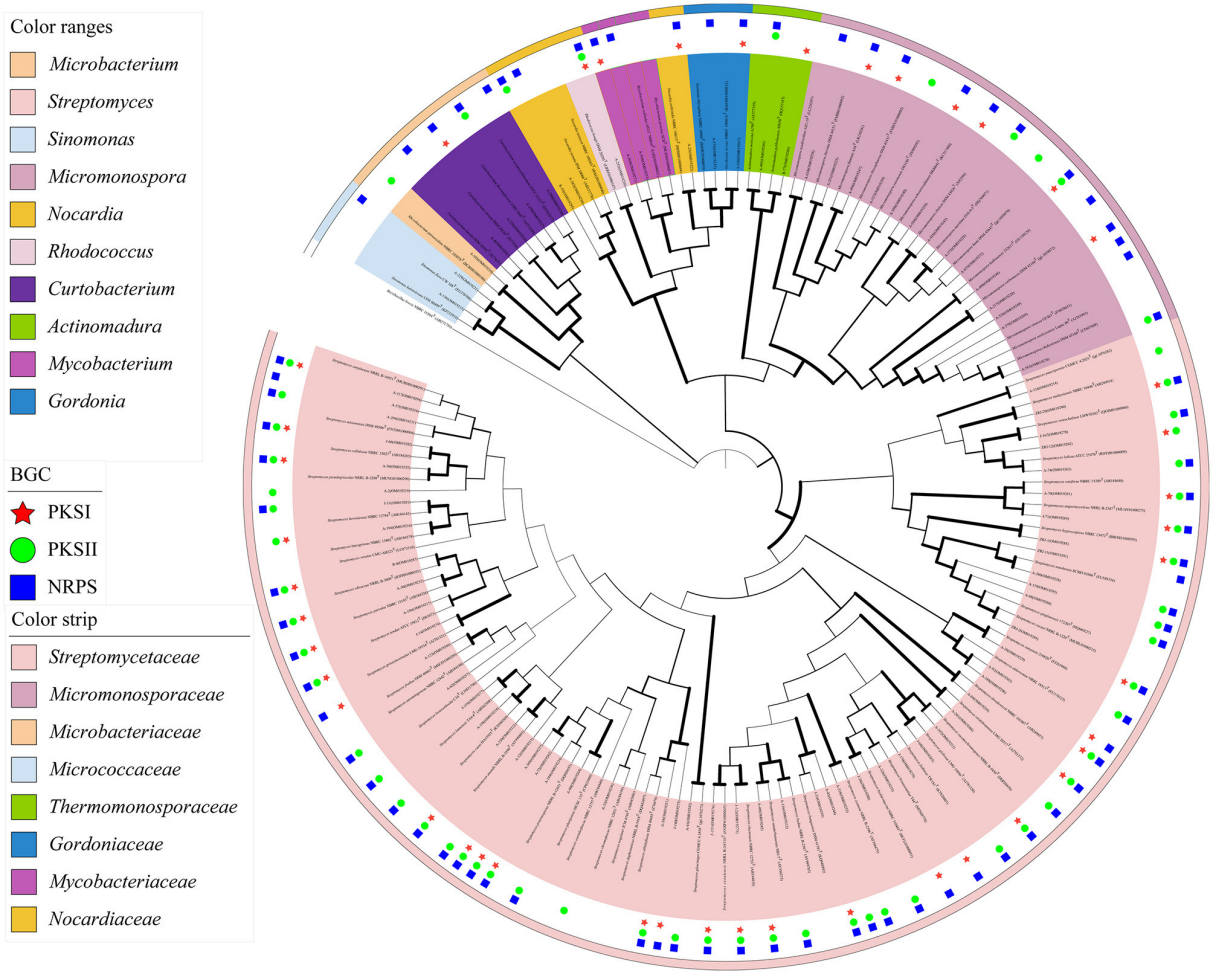
Neighbor-joining phylogenetic tree of 87 representative isolates from mangrove rhizosphere soils in Hainan Island based on 16S rRNA partial gene sequences. Biogenetic gene cluster (BGC) distribution was detected by PCR screening. The values at each node represent the bootstrap values from 1,000 replicates. *Brevibacillus brevis* served as an outgroup.

Based on the analyses of partial 16S rRNA sequences and phenotypic characteristics, 87 representative isolates were selected for the phylogenetic analysis utilizing the MEGA 7.0 software. The confidence level was calculated at a 1,000 bootstrap value to visualize the relatedness of the isolates and their closely related actinobacterial species using the neighbor-joining (NJ) algorithm (Figure 2). The actinobacterial species were clustered into 10 genera, namely, *Streptomyces, Micromonospora, Rhodococcus, Sinomonas, Nocardia, Gordonia, Actinomadura, Mycobacterium, Curtobacterium*, and *Microbacterium*, belonging to six orders, *Streptomycineae, Micrococcineaes, Micrococcales, Micromonosporales, Corynebacteriales*, and *Microbacteriales*. The predominant genus was *Streptomyces* (68.29%, 196 strains), followed by *Micromonospora* (16.03%, 46 strains) and *Curtobacterium* (8.36%, 24 strains).

**Figure 3:**
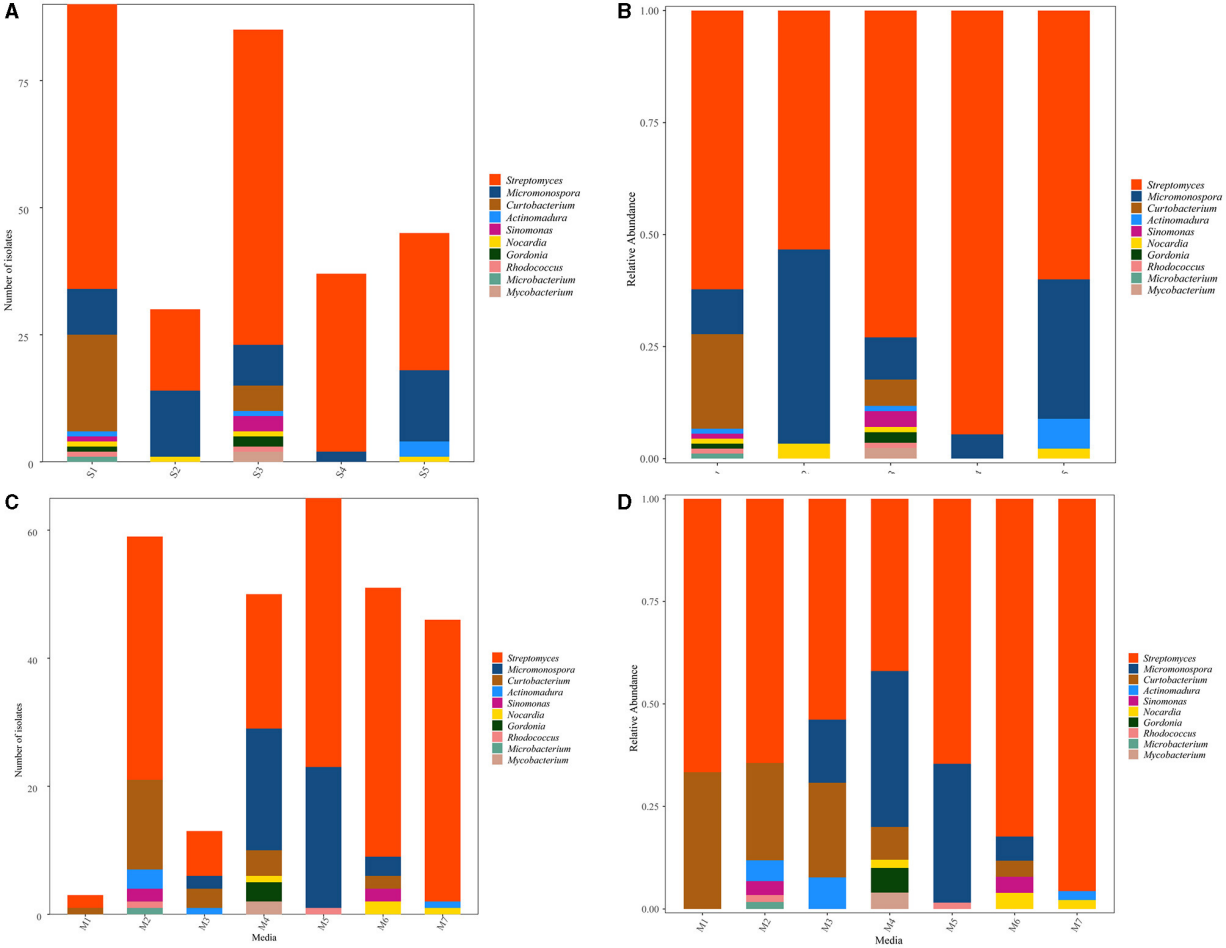
Distribution of culturable Actinobacteria from mangrove rhizosphere soils in Hainan Island. **(A, B)** Taxonomy summary bar plot of genera distribution in different sampling sites. **(C, D)** Summary bar plot of taxonomic distributions at the genera level according to the culture media used for isolation.

The distribution of 287 representative isolates from five sampling sites is displayed in Figures 3A, B. S1 contributed to the highest number and diversity of isolation (90 strains, distributed in nine genera, 31.36%), followed by S3 (85 strains, distributed in nine genera, 29.62%), S5 (45 strains, distributed in four genera,15.68%), S4 (37 strains, distributed in two genera, 12.89%), and S2 (30 strains, distributed in three genera, 10.45%). The heat map (Supplementary Figure 2A) showed that the species affiliated to genera *Streptomyces* and *Rhodococcus* were dominant in S1 and S3; *Curtobacterium* and *Microbacteriumcan* were recovered in S1, while isolates belonging to *Sinomonas, Gordonia*, and *Mycobacterium* were observed in S3. The *Actinomadura* species were the most diverse in S5. *Micromonospora* could be obtained from S4, but not in abundance. *Nocardia* could be obtained from all samples but not in S4.

Among the seven different media used to isolate Actinobacteria, M2 was the most suitable medium for isolating actinobacterial strains due to the number and diversity of obtained isolates that provided 20.56% (59 strains, distributed in six genera) of the total isolates. M4 (50 strains in six genera, 17.42%) and M6 (51 strains in five genera, 17.77%) were the second best, followed by M7 (46 strains in three genera, 16.03%) and M3 (13 strains in four genera, 4.53%). However, M5 yielded the highest number of isolates with low diversity (65 strains in three genera, 22.65%), and M1 yielded the lowest number and variety of isolates (3 strains in two genera, 1.05%), as shown in Figures 3C, D. Concerning the medium composition, it was also found that the species belonged to *Curtobacterium, Microbacterium*, and *Actinomadura* and could be easily isolated from M2. In contrast, Gordonia and *Mycobacterium* could be easily isolated from M4. Actinobacteria genus *Nocardia, Micromonospora, Streptomyces, Sinomonas*, and *Rhodococcus* showed no significant difference in the isolation media (Supplementary Figure 2B).

To better understand the actinobacterial community, isolates from the various sampling sites and isolation culture media were compared using α and β diversity. The α-diversity indices such as Richness (2–9), Shannon (0.21–1.15), Simpson (0.10–0.56), Pielou (0.30–0.67), Invsimpson (1.11–2.26), and Chao1 (2–24) varied significantly among the studied sampling sites (Table 1). The Shannon and Simpson indices showed the highest species richness of the sample collected in S1 (*H'*= 1.15, *1–D* = 0.56), followed by S3 (*H'*= 1.07, *1–D* = 0.45). The uneven Pielou and Invsimpson index indicated variant species composition across different sites. The observed number of actinobacterial isolates in S1 and S3 were still lower than predicted in the estimate using the non-parametric Chao1indice. All indices agreed with the less diverse communities being in S4. The principal coordinate analysis (PCoA) plot revealed differences (β-diversity) in the composition of actinobacterial communities (Supplementary Figure 3A). The first two PCoA axes explained 96.00% of the total variation in the community composition. A similar clustering pattern of S1 and S3 was observable. Further, S2, S4, and S5 appeared to be distributed separately from the other samples.

**Table 1:** α-diversity indices of Actinobacteria recovered from different sampling sites.

	**Richness**	**Shannon**	**Simpson**	**Pielou**	**Invsimpson**	**Chao1**
S1	9	1.15	0.56	0.53	2.26	24
S2	3	0.81	0.53	0.74	2.11	3
S3	9	1.07	0.45	0.49	1.83	10
S4	2	0.21	0.10	0.30	1.11	2
S5	4	0.93	0.54	0.67	2.17	4

In addition, clearly distinct taxonomic and phylogenetic composition was observed in the isolation culture media under study (Table 2). Diversity indices (α-diversity) revealed M4 (*H'* = 1.31, *1–D* = 0.67, Pielou = 0.073, Invsimpson = 3.0) to be suitable for Actinobacteria isolation from rhizosphere soil and more diverse than the others. Unconstrained PCoA showed a distinct clustering pattern, and M2, M6, and M7 clustered together, Bray-Curtis metrics explained 84.49% of the variation (Supplementary Figure 3B).

**Table 2:** α-diversity indices of Actinobacteria recovered from different isolation culture media.

	**Richness**	**Shannon**	**Simpson**	**Pielou**	**Invsimpson**	**Chao1**
M1	2	0.64	0.44	0.92	1.80	2.00
M2	6	1.03	0.52	0.57	2.10	6.50
M3	4	1.16	0.63	0.83	2.68	4.00
M4	6	1.31	0.67	0.73	3.00	6.00
M5	3	0.71	0.47	0.65	1.88	3.00
M6	5	0.71	0.31	0.44	1.46	5.00
M7	3	0.21	0.08	0.19	1.09	4.00

### Antimicrobial potential of actinobacterial isolates

**Figure 4:**
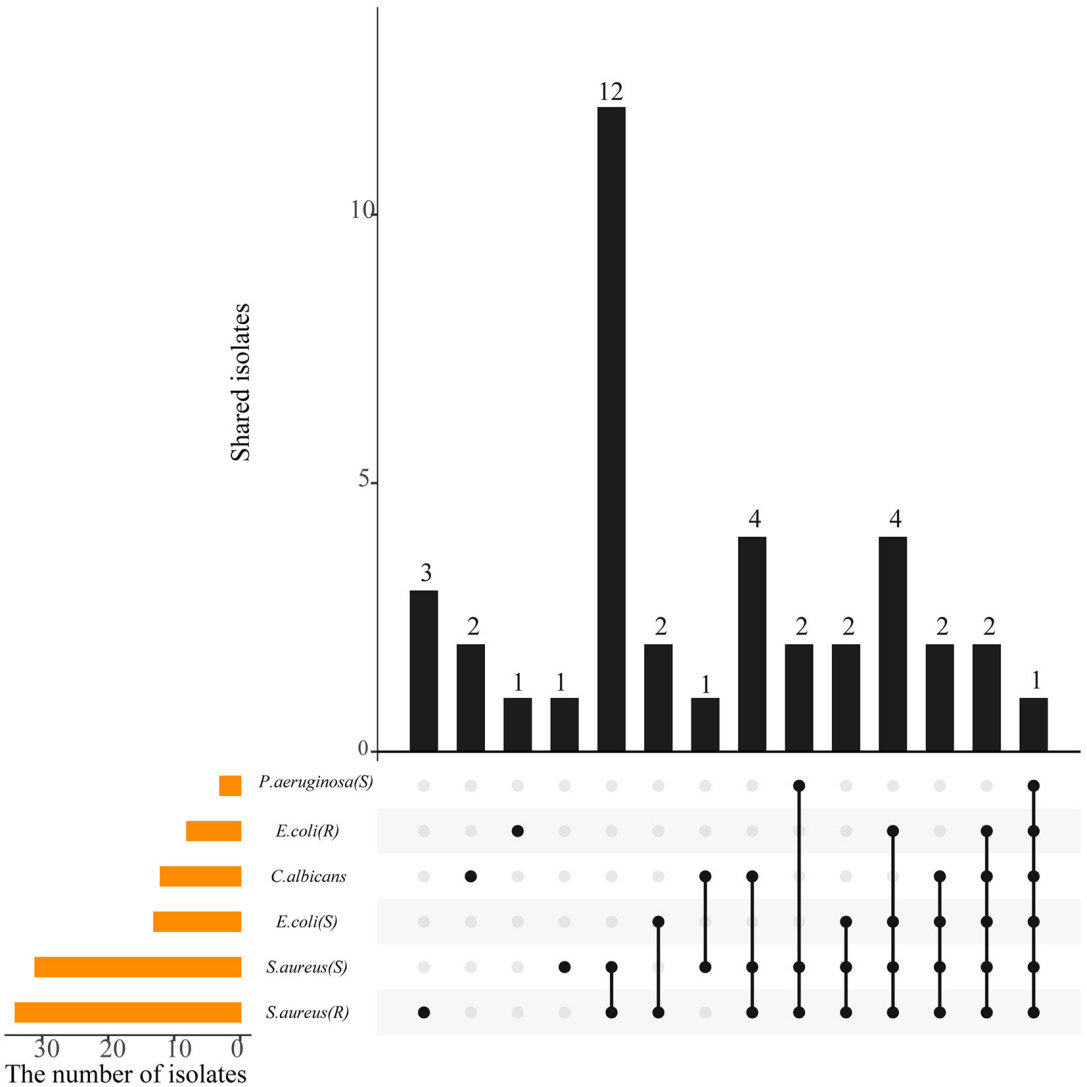
A UpSetR plot showing the shared and unique antimicrobial activity among the actinobacterial isolates. The strip at the bottom left shows the number of isolates that showed inhibition against each indicator microorganism. The dot and line at the bottom right represent the subsets of antimicrobial activities. The number of relevant isolates in each subset is represented in the histogram, which is the upper part of the whole plot.

Crude extracts of 87 representative isolates were subjected to antimicrobial evaluation by determining the MIC of each strain against six indicator microorganisms. Approximately 39 strains (44.83%), affiliated to six genera, including *Streptomyces* (28), *Micromonospora* (5), *Curtobacterium* (3), *Actinomadura* (1), *Sinomonas* (1), and *Gordonia* (1), exhibited antagonistic activity against at least one of the tested pathogen at the selected concentration of 1 mg/mL (P<0.05) (Supplementary Tables 3, 6). The antimicrobial profile of 39 isolates against indicator pathogens is shown in Figure 4. Of the 39 strains, 16 were active only against gram-positive bacteria (*S. aureus*), while one was active only against gram-negative bacteria (*P. aeruginosa* and *E. coli*), and two were active only against *C. Albicans*. Regarding the sensitive pathogenic indicators, the activity against *S. aureus* (ATCC 29213) was clearly the most frequent (31, 35.63%), and activity against *P. aeruginosa* was the least frequent (3, 3.45%), while 14.94% (13) of the isolates were active against *E. coli* (ATCC 25922). As for the resistant pathogenic indicators, activity against *S. aureus* (ATCC 25922) was the most frequent (34, 39.08%), compared to *E.coli* (ATCC 33591; 8, 9.20%). Besides, 12 strains (13.79%) were active against the fungal pathogen *C. albicans*. Additionally, the antimicrobial activity of the same strain was significantly different (*P* < 0.05) in different extract fractions, but all of the antimicrobial activities were weaker than that of the positive control. EF extracts were determined to be more suitable for antibiotic production than MF and WF. EF from the actinobacterial isolates, A-30 (*Streptomyces parvulus*), A-326 (*Streptomyces broussonetiae*), J-151 (*Streptomyces seoulensis*), R-77 (*Actinomadura geliboluensis*), and ZRJ-11 (*Streptomyces qinglanensis*) was most potent against *S. aureus* and its resistant strain with MIC values reaching 7.8 μg/mL, compared to the clinical antibiotic ciprofloxacin (MIC = 3.9 μg/mL). Of them, strain A-30 showed the broadest antimicrobial spectrum against six indicator test microorganisms. A broad spectrum of antimicrobial activities was also obtained with EF of several *Streptomyces* isolates, A-56 (*S. pseudogriseolus*), A-70 (*S. caniferus*), A-107 (*S. racemochromogenes*), A-189 (*S. bingchenggensis*), A-326 (*S. broussonetiae*), ZRJ-1 (*S. hygroscopicus*), J-151 (*S. seoulensis*), and J-170 (*S. corchorusii*) and were active against four or five indicators. Notably, some rare actinobacterial isolates, such as A-498 (*Micromonospora haikouensis*) and R-77, exhibited significant inhibition toward gram-positive bacteria, which could be a potential storehouse for novel antibiotics.

### Immunosuppressive potential of actinobacterial isolates

**Figure 5:**
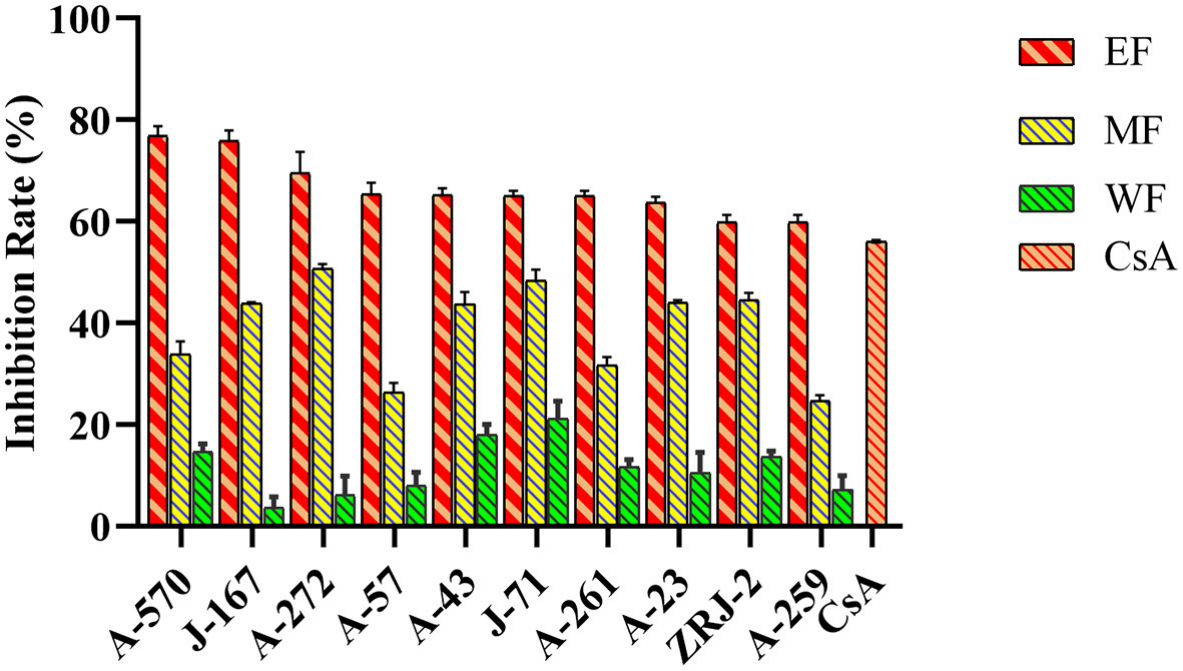
Immunosuppressive activity of actinobacterial isolates from mangrove rhizosphere soils in Hainan Island on the inhibition of Con A-induced murine splenocytes proliferation. EF, Ethyl acetate fraction; MF, Mycelial acetone fraction; WF, Water fraction; CsA, Cyclosporine A.

Representative strains were evaluated for immunosuppressive activities against the proliferation of Con A-induced T murine splenic lymphocytes *in vitro*. Our results showed that 48 strains (55.17%) affiliated to eight genera, *Streptomyces* (31, 35.63%), *Micromonospora* (7, 8.05%), *Curtobacterium* (3, 3.45%), *Actinomadura* (2, 2.30%), *Gordonia* (2, 2.30%), *Sinomonas* (1, 1.15%), *Nocardia* (1, 1.15%), and *Rhodococcus* (1, 1.15%), displayed different levels of inhibitory activity against T cells growth at 10 μg/mL extracts (Figure 5, Supplementary Tables 4, 6). Additionally, EF extracts showed higher inhibitory capacities than the other two extraction fractions. Interestingly, among all the active actinobacterial strains, 10 isolates exhibited potent immunosuppressive activity with an inhibition rate higher than 61.77%, including the rare Actinobacteria A-570 (*Gordonia rhizosphera*), A-272 (*Micromonospora maritima*), A-23 (*Nocardia arthritidis*), and A-259 (*Curtobacterium flaccumfaciens*), which showed an inhibition rate of 76.84, 69.57, 63.75, and 61.77%, respectively. EF extracts of the isolates had the highest occurrence of immunosuppressive activity compared with those of other fractions from the water phase or mycelium. Cyclosporine A was used as the positive control, and the inhibition rate was 56.08%. Notably, the 10 Actinobacteria were stronger than the inhibition rate of cyclosporine A, suggesting that these actinobacterial species could have high potential as producers of immunosuppressive compounds.

### Anticancer potential of actinobacterial isolates

**Figure 6:**
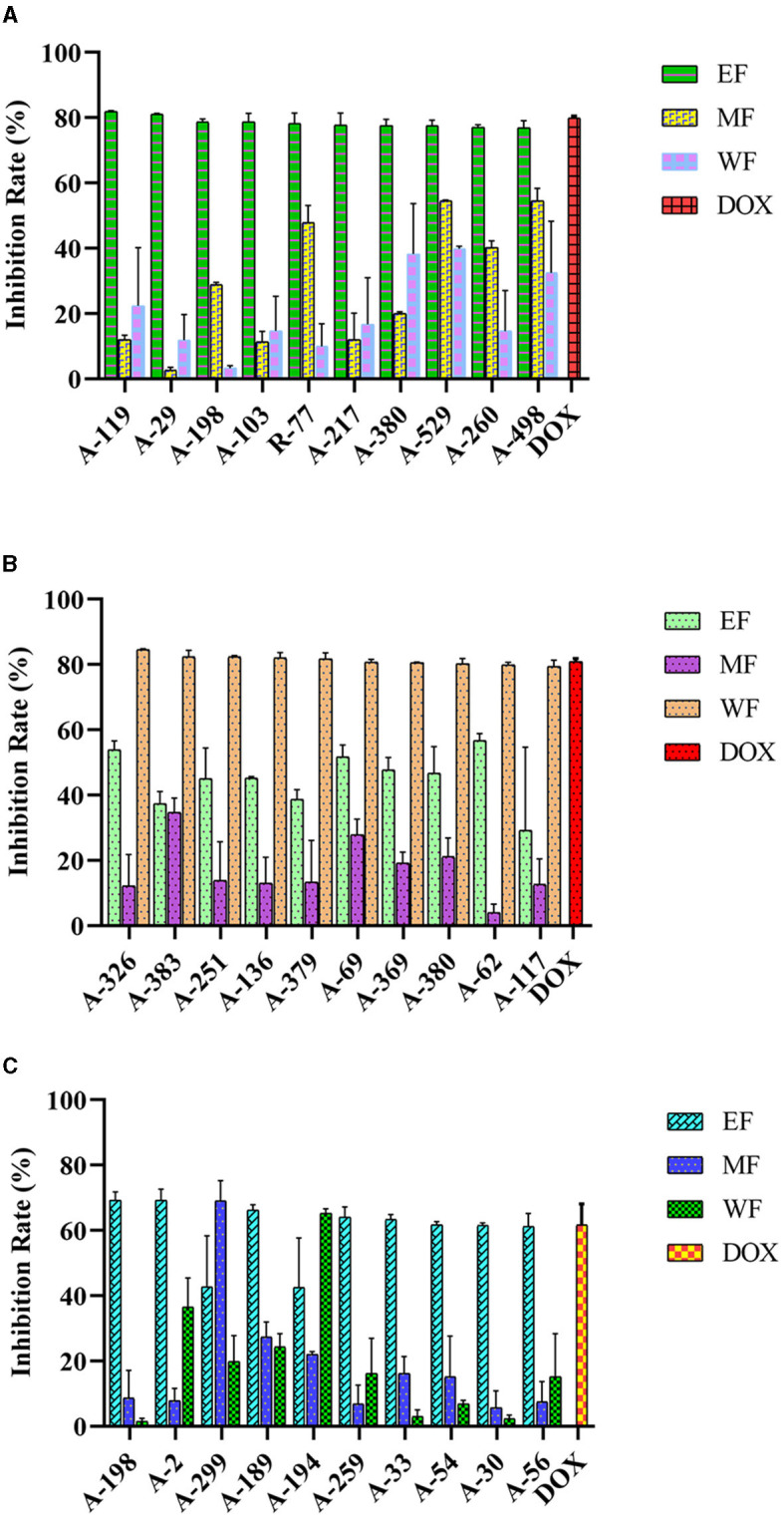
Anticancer activity of actinobacterial isolates from mangrove rhizosphere soils in Hainan Island toward different human cancer cell lines. **(A)** HepG2 **(B)** HeLa, and **(C)** HCT-116. EF, Ethyl acetate fraction; MF, Mycelial acetone fraction; WF, Water fraction; DOX, Doxorubicin.

The anticancer activities of the actinobacterial crude extracts were determined on human cancer cell lines HepG2, HeLa, and HCT-116 using an MTT colorimetric assay. Out of the 87 isolates, 79 strains (90.80%) tested inhibited the cancer cell's proliferation to some extent. They exhibited a percentage of viability cell value ≤50% at a concentration of 10 μg/mL extracts (Figure 6, Supplementary Tables 5, 6). These bioactive strains were distributed in 10 genera, covering *Streptomyces* (50, 57.47%), *Micromonospora* (13, 14.94%), *Curtobacterium* (4, 4.60%), *Nocardia* (3, 3.45%), *Sinomonas* (2, 2.30%), *Gordonia* (2.30%), *Mycobacterium* (2, 2.30%), *Actinomadura* (1,1.15%), *Rhodococcus* (1, 1.15%), and *Microbacterium* (1,1.15%), suggesting the cytotoxic potential of these actinobacterial isolates. In general, 13 extracts (14.94%) were effective against all three tested cancer cell lines, most obtained from the isolates belonging to the genus *Streptomyces* and none from *Micromonospora, Rhodococcus, Microbacterium, Actinomadura*, and *Mycobacterium*. The majority of extracts, 78.16% (68 strains), showed cytotoxicity and displayed a HepG2 cell viability percentage ≤50%. Of these, 10 EF extracts exhibited significant cytotoxicity against HepG2 cells with an inhibition rate over 76.89%, including six rare Actinobacteria A-103 (*Microbacterium paraoxydans*), R-77 (*Actinomadura geliboluensis*), A-217 (*Micromonospora carbonacea*), A-529 (*Micromonospora saelicesensis*), A-260 (*Gordonia terrae)*, and A-498 (*Micromonospora haikouensis*), with an inhibition rate of 78.67, 78.31, 77.78, 77.59, 77.05, and 76.89%, respectively. Meanwhile, 66.67% (58 strains) of the extracts resulted in cell death of more than 50% of HeLa cells, with 10 water extracts showing strong cytotoxic effects against the HeLa cell line with an inhibition rate of more than 79.38%. In particular, four rare Actinobacteria A-383 (*Nocardia testacea*), A-251 (*Micromonospora chersina*), A-136 (*Sinomonas halotolerans*), and A-379 (*Micromonospora vinacea*) showed the highest activity suppressed 82.38, 82.35, 82.02, and 81.81% of the HeLa cells proliferation. In addition, extracts of 21.84% (19 strains) tested and exhibited cytotoxic effects against HCT-116 cells, among which 7 EF extracts could significantly antiproliferative on HCT-116 cells with an inhibition rate over 61.22%, while including the rare actinobacterial strain A-259 (*Curtobacterium flaccumfaciens*, inhibition rate = 64.03 %). Doxorubicin was used as the positive control, and the inhibition rate were 79.93%, 80.85%, and 61.74% for HepG2, HeLa, and HCT-116 cells, respectively. Notably, EF extracts of two *Streptomyces* species (A-119 and A-29) and WF extracts of five isolates (A-326 *S. broussonetiae*, A-383, A-251, A-136, and A-379) were stronger than the cytotoxicity of doxorubicin toward HepG2 and HeLa, respectively, suggesting that these seven actinobacterial species could have high potential as producers of anticancer compounds.

### Biosynthetic gene clusters screening

To investigate the mangrove rhizosphere soil-derived actinobacterial isolates for their potential to produce secondary metabolites, we detected the conserved regions of BGCs through PCR-based screening targeting the KS domain of PKS-I, KSα domain of PKS-II and AD domain of NRPS. In total, 86 (98.90%) of 87 strains showed an unambiguous amplicon band of the expected size in the agarose gel for at least one of three targeted genes (Supplementary Table 6, Supplementary Figures 4, 5). More specifically, positive amplification products of the NRPS gene were detected in 77 strains (88.51%), while PKS II and PKS I genes were detected in only 58 (66.67%) and 43 (49.43%) strains. In addition, 66 strains showed positive results for at least two of three targeted genes, and 26 strains possessed three types of biosynthesis genes simultaneously (Supplementary Figure 4). In contrast to the genus *Streptomyces*, PKS-I/II and NRPS genes were seldom detected at the same time in rare Actinobacteria studied except for two strains, A-635 (*Micromonospora humi*) and A-252 (*Rhodococcus hoagii*).

### Bioactivity correlation with biosynthetic gene clusters

**Figure 7:**
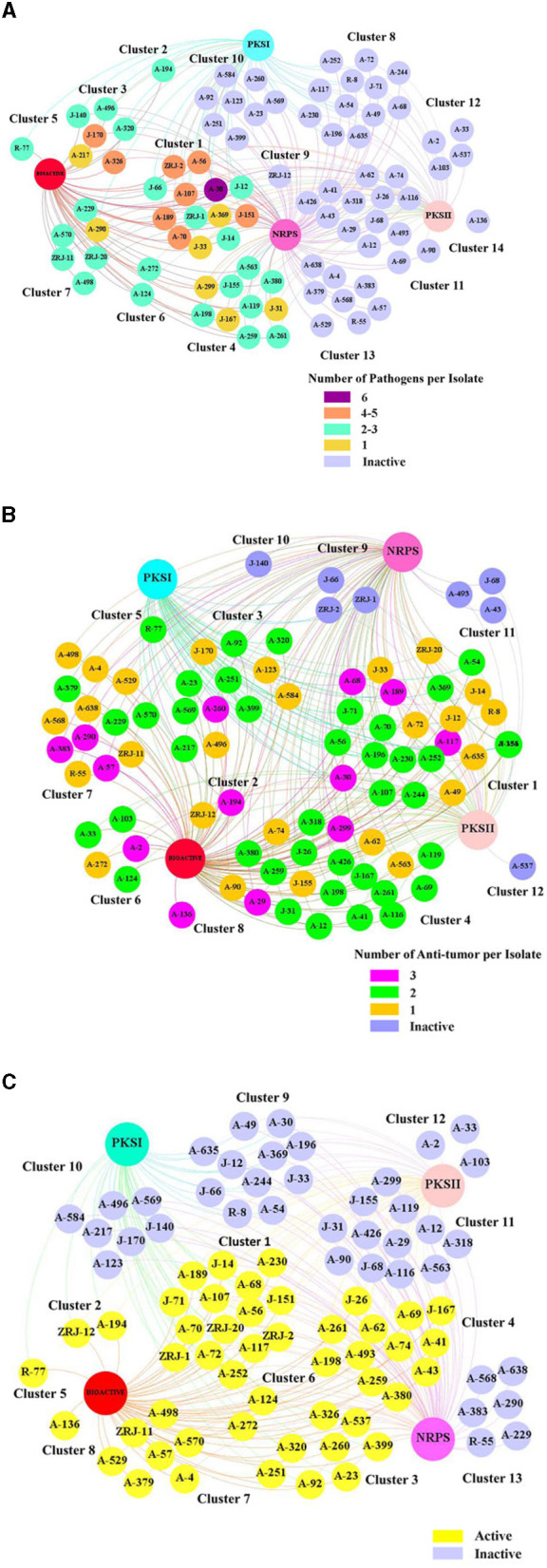
Correlation network of the bioactivity capacity of 87 mangrove rhizosphere actinobacterial isolates and their biosynthetic gene clusters (BGCs). The big nodes represent the BGCs. The 87 isolates are presented in small nodes with their corresponding isolate code. The color of each node indicates whether each strain was active or inactive. **(A)** Antimicrobial activity: The number of susceptible pathogens against each isolate was correlated with the number of BGCs detected in their genome. The color of each node indicates the number of pathogens that were inhibited by each isolate. **(B)** Anticancer activity: The number of human cancer cells against each isolate was correlated with the number of BGCs detected in their genome. The color of each node indicates the number of human cancer cells that were inhibited by each isolate. **(C)** Immunosuppressive activity: The effect of isolates inhibiting the proliferation of splenocytes induced by Con A was correlated with the number of BGCs detected in their genome.

With the confirmation of multiple BGCs in 86 of the representative 87 isolates, we hypothesized that bioactivities were positively correlated with the number of BGCs present in its genome. Thus, type I and II PKS and NRPS were associated with active and inactive actinobacterial isolates. Relationships formed were visualized through “Gephi.” The network visualization showed that 87 mangrove rhizosphere actinobacterial isolates aggregated into 14 major clusters based on their identified BGCs and antimicrobial activity, regardless of the number of susceptible pathogens (Figure 7A). We observed that the active and inactive *Streptomyces* isolates were distinctively separated into different groups. Among the 39 active strains, 14 isolates (35.90%) had NRPS, type I and type II PKS (Cluster 1), 10 isolates (25.64%) lacked type I PKS (Cluster 4), six isolates (15.38%) lacked type II PKS (Cluster 3), one isolate lacked NRPS (Cluster 2), and eight isolates (20.51%) had only contain one type of biosynthetic sequences (Clusters 5–7). Interestingly, active isolates that harbored all three genes (Cluster 1) expressed different bioactivity strengths. A-369 (*Streptomyces prasinosporus*) and J-33 (*S. griseoruber*) were only active against one pathogen. On the contrary, A-30 (*Streptomyces parvulus*) showed the broadest antimicrobial spectrum against six indicator test microorganisms. Furthermore, 12 isolates (Cluster 8) did not elicit antimicrobial activity against six tested pathogens despite their genomes harboring type I and type II PKS and NRPS.

Similarly, a relationship analysis between anticancer activity and BGCs of 87 rhizosphere actinobacterial isolates showed aggregation of twelve major clusters (Figure 7B). Even 23 (29.11%) of 79 active isolates harboring type I and type II PKS and NRPS (Cluster 1), 21 isolates (26.58%) harboring type II PKS and NRPS (Cluster 4), a rare actinobacterial species A-136 (*Sinomonas halotolerans*; Cluster 8) lacked all BGCs and yet were effective against all of the three tested cancer cell lines and all eight inactive stains (Clusters 9–12) harboring at least one type of BGCs were devoid of any anticancer activity.

Furthermore, the relationship analysis of 87 rhizosphere actinobacterial isolates' immunosuppressive activity and BGCs showed different clusters of active and inactive strains (Figure 7C). The 48 active strains were further clustered according to the BGCs present in their genome, which revealed that 12 isolates (25%) with no BGCs (Cluster 8) or with only one BGCs (Clusters 5–7) still displayed different levels of inhibitory activity against T cells growth. Three correlation analyses indicated that the number of BGCs present in an isolate did not necessarily dictate its antimicrobial, anticancer activity, or immunosuppressive capacity and vice versa, contrary to our initial hypothesis.

## Discussion

The mangrove ecosystems support biologically different organisms and microbes, accounting for 60–75% of the biomass in tropical and subtropical coastal areas (Zhang and Liao, 2022). Mangrove Actinobacteria are of particular interest as they make up only a small portion (4%) of microbial species originating from the mangrove ecological niche at the domain level. Still, they are capable of producing chemically diverse compounds with an extraordinarily high proportion (60%) of their metabolites showing a wide range of biological activities (Xu, 2015). Although mangrove rhizosphere soil-derived Actinobacteria have been continuously reported as an expected wellspring of bioactive secondary metabolites for pharmaceutical aspects entirely different from their terrestrial counterparts (Li et al., 2022), renewed efforts in isolating these strains and their biological or metabolic biosynthetic potential are ongoing. The present study applied an integrated strategy of combining phylogenetic diversity, biological activities, and biosynthetic gene clusters (BGCs) screening approach to investigate Actinobacteria isolated from mangrove rhizosphere soils from Hainan Island. The results showed that the actinobacterial community was highly diverse, and a total of 287 independent isolates affiliated to 10 genera in eight families of six orders were recognized out of five geographical regions by using seven selective isolation media. The genera *Streptomyces* and *Micromonospora* still accounted for the predominant numbers of the isolates, a finding in line with previous studies. For instance, Zhao et al. (2022) reported 49 Actinobacteria belonging to six genera from rhizosphere sediments of three mangrove plants with the dominant genera *Streptomyces*. A total of 88 Actinobacteria strains affiliated to nine genera in eight families in six orders were isolated from eight different mangrove rhizosphere soil samples of Maowei Sea in Guangxi Province with the dominant genera *Streptomyces* and *Micromonospora* (Ye et al., 2018). Based on this study and other reports (Wang et al., 2014), some of the mangrove rhizosphere soil Actinobacteria were related to common genera in terrestrial habitats. For example, the *Streptomyces parvulus* strain in the rhizosphere soil of mangroves is thought to be closely related to the *Streptomyces parvulus* strain obtained from the soil of the Yellow River Delta, with a homology of 99%. No direct evidence suggested the presence of obligated Actinobacteria in the mangrove rhizosphere soil of the Hainan Islands, indicating that these isolates were maybe facultative Actinobacteria of terrestrial origin. However, many novel natural products that are not found in terrestrial strains have been found in these mangrove rhizosphere soil Actinobacteria (Zeng et al., 2013; Xu et al., 2014; Jose et al., 2021), suggesting these Actinobacteria have adapted to the special environment and have their unique metabolic activities.

On further comparison of the actinobacterial community structure, clearly distinct taxonomic and phylogenetic compositions were found between the counts isolated from different geographic locations and isolation media. Previous phylogenetic studies have suggested that the mangrove actinobacterial community composition was best predicted by differences in geographical location, pH, temperature, salinity, moisture, and nutrient and organic matter in sediments and seawater (Amrita et al., 2012). The diversity indices showed the highest species richness of the sample collected in S1 (sampled surrounding *Ceriops tagal* in May 2021), followed by S3 (sampled surrounding *Rhizophora apiculata* in March 2021). All indices agree with the less diverse communities being in S4 (sampled surrounding *Rhizophora stylosa* in January 2021). This may be because Actinobacteria present in the rhizosphere soil can be transferred easily between the plant roots, allowing the plant to determine the composition of its bacterial community (Liu J. W. et al., 2021). Furthermore, the actinobacterial community composition was influenced by precinct environmental heterogeneity and geographic distance, and temperature levels exceeding certain thresholds led to significant changes in the structure of bacterial communities (Liu et al., 2017). Meanwhile, the alteration of isolation media is an essential means to promote the growth of the actinobacterial taxa population in the isolation process (Zhang et al., 2020), and our study testified that the composition of the media had different influences on the number and diversity of Actinobacteria recovered from mangrove rhizosphere soils. Among the seven different media employed to isolate Actinobacteria, Gauze's Medium No.2 (M2) was demonstrated to be the most efficient for isolating actinobacterial strains to provide 20.56% (59 strains, distributed in six genera) of the total isolates. The traditional ISP2 (M1) medium has been previously described as an efficient medium for isolating *Streptomyces* species from the soil. In contrast, it did not present an advantage in our study and only allowed retrieval of three actinobacterial strains, but again *Streptomyces* were predominant (Li F. N. et al., 2019). Interestingly, some actinobacterial strains grew exclusively on one medium, such as isolates from the genera *Gordonia* and *Mycobacterium* on Czapek' Medium (M4) and the genera *microbacterium* on Gauze's Medium No. 2 (M2). However, < 5% of all known Actinobacteria were readily cultivable using conventional culture-based techniques, and the rest remain unexplored (Santoferrara et al., 2014). Recent advances in molecular techniques have revealed a “not yet cultured” diversity within this group. Culture-independent Actinobacteria were not considered in the present study. Culture-free methods, including metagenomics analysis, sampling total DNA directly from the environment, and the heterologous expression of a biosynthetic gene in yeast or bacteria, may be adopted as a complementary approach to conventional cultivation, which is essential to describe the diversity and ecology of actinobacterial communities (Krug and Müller, 2014; Arakawa, 2018; Ahmed et al., 2020; Lee et al., 2020; Xu et al., 2022).

Traditionally, selection based on phylogeny (16S rRNA sequences) is useful as a guide to select strains from a specific genus or species of particular interest or even to discard redundant isolates or those pathogenic strains from a collection. Nevertheless, this taxonomical data alone does not provide biological or metabolic information (Betancur et al., 2017). The members of Actinobacteria were generally associated with their antimicrobial properties. However, numerous studies documented that mangrove soil-derived Actinobacteria has become a hot spot for natural product discovery. An impressive array of secondary metabolites with various interesting bioactivities were reported amongst 35.6% of compounds that displayed cytotoxicity, followed by antimicrobial (9.8%), enzyme inhibitory (5.9%), antiviral (4.9%), and anti-oxidative (4.5%; Li et al., 2022) characteristics. This indicated that screening with multiple bioactivity models can constitute an efficient way to improve sensitivity for various diseases detection simultaneously and accelerate the drug discovery process, as the discovery of novel bioactive natural products is encouraged not only by the quality of biological material but also by the innovation and sensitivity of the screening models used.

In the present study, the crude extracts of 87 representative isolates affiliated with 10 genera with unique colony morphology were selected to evaluate their biological potential. The ISP2 medium was reported to support the growth of most Actinobacteria and was known to promote secondary metabolite production (Tata et al., 2019); it was the only medium used for fermentation. After multiple bioactivity screenings among the isolates, many prominent candidates that deserved further exploitation were identified. Among them, 39 strains (44.83%) affiliated to six genera exhibited antimicrobial activity, while 79 strains (90.80%) affiliated to 10 genera showed anticancer activities, and 48 strains (55.17%) affiliated to eight genera were immunosuppressive.

The identification of *Streptomyces* as the most bioactive genus in this study is in line with other research (Hong et al., 2009; Li et al., 2021; Lu et al., 2021) as *Streptomyces* is the largest genus of the Actinobacteria and it can catabolize a wide range of compounds and produce secondary metabolites with diverse biological activities. It is reported that 45% of the presently known bioactive microbial metabolites are still isolated from various actinobacterial species. The *Streptomyces* species produces 7,600 compounds (74% of all Actinobacteria) and represents the largest group of biomolecules (Xu, 2011, 2015). The bioprospecting studies of *Streptomycetes* from mangrove areas are generating fruitful outcomes. It has great capability to produce secondary metabolites such as antibiotics chalcomycin B (Asolkar et al., 2002), and sporeamicin D (Sun et al., 2017), anti-HIV agent xiamycin (Ding et al., 2010), anticancer agents such as streptocarbazoles A and B (Fu et al., 2012), streptomyceamide C (Fu et al., 2016), and neoantimycins A and B (Hu et al., 2017).

Our results showed that EF of the *Streptomyces* isolates, A-30, A-326, J-151, and ZRJ-11 exhibited potent antimicrobial activity compared to clinical antibiotic ciprofloxacin; EF extracts of two *Streptomyces* species (A-119 and A-29) and WF extracts of A-326 were more potent than the cytotoxicity of doxorubicin toward HepG2 and HeLa; EF of six *Streptomyces* species, J-167, A-57, A-43, J-71, A-261, and ZRJ-2 exhibited potent immunosuppressive activity against the proliferation of Con A-induced T murine splenic lymphocytes.

Rare Actinobacteria species obtained from mangrove soil are also proficient producers of bioactive metabolites and drug-like molecules. A potent anticancer agent, salinosporamide A was characterized by *Salinispora tropica* CNB-392 and is currently in phase III clinical trials for treating a series of different cancer types (Feling et al., 2003; Lee and Jeong, 2020). Nocardichelins A and B produced by *Nocardia* strain Acta 3026 strongly inhibited a panel of human cell lines, such as gastric adenocarcinoma, breast carcinoma, and hepatocellular carcinoma cells with GI_50_ values in the low micromolar to nanomolar range (Schneider et al., 2007). Fungicide antimycin A_18_, with a higher activity against plant pathogenic fungi than blasticidin S (a commercialized fungicide), was isolated from a mangrove-derived *Streptomyces albidoflavus* I07A-01824 (Yan et al., 2010). Rifamycin S and its geometric isomer are both antibiotics and have been found co-isolated from an unusual actinomycete species named *Micromonospora rifamycinica* strain AM105 (Huang et al., 2009). Notably, some rare genus species displayed extraordinary biological potential, including antimicrobial strains A-498 (*Micromonospora haikouensis*) and R-77 (*Actinomadura geliboluensis*), anticancer strains A-383 (*Nocardia testacea*), A-251 (*Micromonospora chersina*), A-136 (*Sinomonas halotolerans*), and A-379 (*Micromonospora vinacea*), and immunosuppressive strains A-570 (*Gordonia rhizosphera*), A-272 (*Micromonospora maritima*), A-23 (*Nocardia arthritidis*), and A-259 (*Curtobacterium flaccumfaciens)*. EF of the J-140 (*Streptomyces albidoflavus*) exhibited antimicrobial activity, showing the strengths of this multiple biological screening approach to guide isolation efforts on unknown compounds and the capabilities to identify new sources of known active compounds. The strong inhibitory activities of these strains against a variety of pathogens, cancer cells, and Con A-induced T murine splenic lymphocytes are a good indicator that these isolates could be potential candidates for the production of corresponding bioactive secondary metabolites. Four rare strains, A-136 (*Sinomonas halotolerans*), A-252 (*Rhodococcus hoagii*), A-260 (*Gordonia terrae*), and A-290 (*Curtobacterium oceanosedimentum*) showed good antitumor activity in three tumor cells. Recently, mangromicins, a group of new secondary metabolites with unique chemical structures, were found from *Lechevalieria aerocolonigenes* K10-0216 isolated from a mangrove sediment sample by Omura s group (Nakashima et al., 2015), which further indicated the rare Actinobacteria deserve to be studied extensively and on priority to identify new antibiotics.

In general terms, we propose that each set of data alone is not enough to decide which strains are suitable to study since the bioactivity data alone does not provide information about the chemical compounds responsible for it and can take to the selection of well-known strains producers of well-known compounds (Jose et al., 2021). To overcome this problem, recently, screening and analysis of functional genes based on PCR detection of BGCs that integrates bioactivity information have been described as a useful tool to assess the biosynthetic potential of the culturable or unculturable isolates (Jose and Jha, 2017); for instance, six polyene-polyol macrolides reedsmycins A-F were obtained from mangrove-derived *Streptomyces* sp. CHQ-64 by genome scanning guided isolation (Che et al., 2015). To the best of our knowledge, the majority of compounds from Actinobacteria are demonstrated to be complex polyketides and non-ribosomal peptides (Peng et al., 2020). It is proposed that the detection of BGCs encoding polyketide synthases (PKS I and II) and non-ribosomal peptide synthases (NPRS) can be an efficient way to predict the type of natural products that can be assembled using PKS and NPRS machinery. In the current study, PCR screening of 87 representative isolates revealed that 86 (98.90%) isolates positively hit at least one type of biosynthetic sequence. Meanwhile, 26 strains simultaneously possessed three types of biosynthesis genes, indicating genes associated with PKS/NRPS biosynthesis were widespread and generally distributed in rhizosphere soils derived from Actinobacteria of mangrove.

The percentage of bioactive isolates (44.83% antimicrobial, 90.80% anticancer, and 55.17% immunosuppressive) detected in this study was relatively low compared to the percentage of isolates with at least one type of biosynthetic sequences (98.90%). Most of the isolates showed the presence of genes that encoded PKS/NRPS enzymes, which also showed antimicrobial, anticancer activity, or immunosuppressive capacity. However, eight inactive strains harbored at least one type of BGCs, whereas isolate A-136 lacked all BGCs and yet exhibited significant anticancer activity. Twelve isolates with none or only one BGC present still displayed different levels of immunosuppressive activity. The low bioactivity correlation with biosynthetic gene clusters of the isolates was consistent with the findings of a subset of the researchers (Wu et al., 2017; Li et al., 2021; Sabido et al., 2021). These results indicated that the detected NRPS or PKS gene fragments do not directly imply the production of secondary metabolites; some secondary metabolite biosynthetic gene clusters appear non-active or silent under the fermentation media (ISP2) we used. This was in accordance with a previous study by Schneemann et al. (2010), where ten strains yielded PCR products with PKS-specific primers, but no corresponding PKS products were detected under laboratory conditions (Schneemann et al., 2010). This was similar to some *Streptomyces* species, whose biosynthetic repertoire possessed more than 20 known or predicted genomic regions encoding biosynthetic pathways. Still, only three to five secondary metabolites were isolated, which indicated that those compounds represent only a fraction of the biosynthetic capacity predicted from their genetic information (Sabido et al., 2021). Nevertheless, multiple BGCs in active and inactive strains demonstrated their genetic capacity to produce useful secondary metabolites if cultivated under the appropriate conditions or encountered with specific triggers to induce these silent or cryptic biosynthetic pathways.

To increase the diversity of available actinobacterial secondary metabolites, the OSMAC (one strain of many compounds) approach, developed by Zeeck in the early 2000's, has been demonstrated to be a simple and effective method (Bode et al., 2002). This approach activated the cryptic BGCs by changing medium composition and cultivation status or co-cultivation with other strain(s). The alteration of the media component had a noticeable impact on obtaining new natural products from *Streptomyces* through the OSMAC approach (Nguyen et al., 2020). The production of bioactive metabolites can also be significantly enhanced by rewiring the regulatory network, such as the engineering of promoters, regulatory factors, and ribosomes (Liu Z. Y. et al., 2021). For instance, to explore the potential biosynthetic ability of the *Streptomyces* sp. CHQ-64 mentioned above, a reedsmycins non-producing mutant strain *Streptomyces* sp. CHQ-64Δ*rdmF* was obtained by knocking out the positive regulatory gene *rdmF* involved in reedsmycins biosynthesis, which led to the isolation of two originally cryptic alkaloids, geranylpyrrol A and piericidin F (Han et al., 2017). The absence of amplification products from some isolates may reveal the lack of PKS-I, PKS-II, and NRPS genes, such as isolate A-136. However, the negative results could be caused by the types of degenerate primers used, which were not suitable for amplifying these genes. This opinion was similar to the studies of Peng et al. (2020), where strain SYP-A7257 exhibiting no NRPS amplicons produced actinomycin derivatives synthesized via an NRPS biosynthetic gene cluster. Hence, despite being favorable for the great majority of known PKS/NRPS genes, the chosen primer systems used in this study may not work in all cases of polyketide and non-ribosomal peptides with uncommon molecular constructions. Therefore, the molecular screening of actinobacterial isolates for genes encoding biosynthesis of bioactive compounds is still an effective and valuable approach for preselecting isolates for useful secondary metabolite production.

## Conclusion

In conclusion, this study demonstrated that Actinobacteria from mangrove rhizosphere soil in Hainan Island are underexplored sources of antimicrobial, anticancer, and immunosuppressive agents concerning their metabolic potential for polyketides and non-ribosomal peptides. A total of 287 culturable actinobacterial isolates of at least 10 genera within 8 families of 6 orders were revealed. Of 87 representative strains selected for biological activities, the PKS/NRPS screening indicated “*Streptomyces*-*Micromonospora*” as the major group and demonstrated high biosynthetic potential. Using cues from the current investigation, these biosynthetic Actinobacteria derived from mangrove rhizosphere soil, along with those that displayed bioactivity, should be given priority for the exploration of the corresponding structurally interesting biological natural products.

## Data availability statement

The original contributions presented in the study are included in the article/Supplementary material, further inquiries can be directed to the corresponding author/s.

## Author contributions

JX conceived the study, designed the experiments, did the final revision of the manuscript, supervision, project administration, and funding acquisition. R-jZ and N-lW collected the samples. J-jY and R-jZ did the phylogenetic diversity, biosynthetic gene cluster screening, formal analysis, and data curation. R-jZ did the antimicrobial assay. D-dZ and D-dC performed the anticancer assay. X-lD and R-jZ did the immunosuppressive assay. J-jY did validation, writing of the original draft, and visualization. All authors have read and agreed to the published version of the manuscript.
