# Supplementary material for: Insights into the phylogenetic diversity, biological activities, and biosynthetic potential of mangrove rhizosphere Actinobacteria from Hainan Island
Source: Front Microbiol. 2023 May 31;14:1157601. doi: 10.3389/fmicb.2023.1157601 (PMC10264631; doi:10.3389/fmicb.2023.1157601)
Supplement: Supplementary file 1 [file Data_Sheet_1.docx]

***Supplementary Material***

**Table S1 Information of samples collected from mangrove plant rhizosphere soils**

| Sample number  number | Sample type or Plant species | Site information | Collection date |
| --- | --- | --- | --- |
| 1 | *Ceriops tagal* | N:19°58′19″ E:110°33′50″ | 2021.05 |
| 2 | *Rhizophora apiculata* | N:19°37′45″ E:110°50′5″ | 2021.03 |
| 3 | *Rhizophora apiculata* | N:19°37′40″ E:110°50′6″ | 2021.03 |
| 4 | *Rhizophora stylosa* | N:19°57′14″ E:110°35′10″ | 2021.01 |
| 5 | *Rhizophora stylosa* | N:20°0′6″E:110°33′2″ | 2021.05 |

**Table S2 Compositions of seven different media used for isolation of mangrove endophytic Actinobacteria**

| **No.** | **Name** | **Composition (In 1.0 L distilled water)** |
| --- | --- | --- |
| M1 | ISP 2 medium | Yeast extract 4.0 g, Glucose 4.0 g, Malt extract 5.0 g, Vitamin mixture 1.0 mL, Trace salt 1.0 mL, Agar 20.0 g, pH 7.2 |
| M2 | Gauze’s No.2 medium | Tryptone 3.0 g , Glucose 10.0 g, NaCl 5.0 g , Peptone 5.0 g, Vitamin mixture 1.0 mL, Agar 20.0g, pH 7.2 |
| M3 | NA medium | Peptone 10.0 g, NaCl 5.0 g, Beef extract 3.0 g,Agar 18.0 g, pH 7.3 |
| M4 | Czapek’medium | NaNO_3_ 3.0 g, K_2_HPO_4_•3H_2_O 1.0 g, MgSO_4_ 0.5 g, KCl 0.5 g, FeSO_4_ 0.01 g, Sucrose 30.0 g, Sea salt 3.0 g, Agar 18.0 g, pH 7.3 |
| M5 | Gauze’s No.1 medium | Sea salt 3.0 g , Soluble starch 20.0g, MgSO_4_ 0.5 g, K_2_HPO_4_ 0.5 g, KNO_3_ 1.0g, Agar 18 g, pH 7.3 |
| M6 | R2A medium | R2A (Haibo Biological Corporation, China) 3.2g, pH 7.2 |
| M7 | SC medium | Soluble starch 10.0 g, Casein 0.3 g , KNO_3_ 1.0 g, NaCl 2.0 g , MgSO_4_ •7H_2_O 0.05 g, K_2_HPO_4_ 2.0 g , CaCO_3_ 0.02 g, FeSO_4_ 0.01 g, Agar 20.0 g, pH 7.3 |

Note: Trace salt solution: FeSO_4_·7H_2_O 0.2 g, MnCl_2_•4H_2_O 0.1 g , ZnSO_4_·7H_2_O 0.1g, Distilled water 1.0 L

Vitamin mixture: Thiamine 0.5 g, Calcium pantothenate 0.5 g, Riboflavin 0.5 g, Niacin 0.5 g, P-Aminobenzyl Alcohol 0.5 g, Pyridoxamine 0.5 g, Inositol 0.5 g, Biotin 0.5 g, Distilled water 1.0 L.

**Table S3 Antimicrobial activities of culturable Actinobacteria from mangrove soil and their similarity of 16S rRNA gene sequences.**

| Isolate(GenBank accession no.) | Genus and species (Similanity,%) | Fractions^a^ | Activity^b^ | | | | | |
| --- | --- | --- | --- | --- | --- | --- | --- | --- |
|  |  |  | *S.aureus*(R) | *S.aureus*(S) | *E.coli*(S) | *E.coli*(R) | *P.aeruginosa*(S) | *C.albicans* |
| A-2  (OM019219)  A-4  (OM019241)  A-12  (OM019213)  A-23  (OM019222)  A-29  (OM019229)  A-30  (OM019232)  A-33  (OM019236)  A-41  (OM019242)  A-43  (OM019244)  A-49  (OM019245)  A-54  (OM019251)  A-56  (OM019252)  A-57  (OM019254)  A-62  (OM019257)  A-68  (OM019260)  A-69  (OM019259)  A-70  (OM019261)  A-72  (OM019262)  A-74  (OM019263)  A-90  (OM019264)  A-92  (OM019265)  A-103  (OM019210)  A-107  (OM019211)  A-116  (OM019212)  A-117  (OM019294)  A-119  (OM019295)  A-123  (OM019266)  A-124  (OM019214)  A-136  (OM019215)  A-189  (OM019296)  A-194  (OM019216)  A-196  (OM019217)  A-198  (OM019218)  A-217  (OM019220)  A-229  (OM019221)  A-230  (OM019223)  A-244  (OM019224)  A-251  (OM019225)  A-252  (OM019226)  A-259  (OM019227)  A-260  (OM019267)  A-261  (OM019268)  A-272  (OM019228)  A-290  (OM019230)  A-299  (OM019231)  A-318  (OM019233)  A-320  (OM019234)  A-326  (OM019235)  A-369  (OM019237)  A-379  (OM019269)  A-380  (OM019238)  A-383  (OM019239)  A-399  (OM019240)  A-426  (OM019243)  A-493  (OM019246)  A-496  (OM019247)  A-498  (OM019248)  A-529  (OM019249)  A-537  (OM019250)  A-563  (OM019270)  A-568  (OM019253)  A-569  (OM019271)  A-570  (OM019255)  A-584  (OM019256)  A-635  (OM019272)  A-638  (OM019258)  J-12  (OM019273)  J-14  (OM019274)  J-26  (OM019280)  J-31  (OM019281)  J-33  (OM019293)  J-66  (OM019282)  J-68  (OM019283)  J-71  (OM019284)  J-140  (OM019275)  J-151  (OM019276)  J-155  (OM019277)  J-167  (OM019278)  J-170  (OM019279)  R-8  (OM019287)  R-55  (OM019285)  R-77  (OM019286)  ZRJ-1  (OM019288)  ZRJ-2  (OM019289)  ZRJ-11  (OM019291)  ZRJ-12  (OM019292)  ZRJ-20  (OM019290)  Ciprofloxacin  Amphotericin B | *Streptomyces venetus* CMU-AB225^T^  (99.6%)  *Curtobacterium luteum* DSM 20542^T^  (99.87%)  *Streptomyces aurantiogriseus* NBRC 12842^T^  (99.2%)  *Nocardia arthritidis* NBRC 100137^T^  (99.07%)  *Streptomyces sanyensis* 219820^T^  (100%)  *Streptomyces parvulus* NBRC 13193^T^  (100%)  *Streptomyces chromofuscus* NBRC 12851^T^  (98.8%)  *Streptomyces glauciniger* CGMCC 4.1858^T^  (99.47%)  *Streptomyces bungoensis* DSM 41781^T^  (99.87%)  *Streptomyces sundarbansensis* MS1/7^T^  (99.73%)  *Streptomyces daghestanicus* NRRL B-5418^T^  (100%)  *Streptomyces pseudogriseolus* NRRL B-3288^T^  (100%)  *Streptomyces carpinensis* NRRL B-16921^T^  (98.54%)  *Streptomyces leeuwenhoekii* C34^T^  (98.55%)  *Streptomyces qinglanensis* 172205^T^  (99.87%)  *Streptomyces drozdowiczii* NBRC 101007^T^  (99.47)  *Streptomyces caniferus* NBRC 15389^T^  (100%)  *Streptomyces nogalater* JCM 4799^T^  (98.68%)  *Streptomyces lydicus* ATCC 25470^T^  (99.73%)  *Streptomyces pluripotens* MUSC 135^T^  (99.87%)  *Streptomyces aldersoniae* NRRL 18513^T^  (99.6%)  *Microbacterium paraoxydans* NBRC 103076^T^  (99.6%)  *Streptomyces racemochromogenes* NRRL B-5430^T^  (99.6%)  *Streptomyces badius* NRRL B-2567^T^  (99.73%)  *Streptomyces malaysiense* MUSC 136^T^  (100%)  *Streptomyces qinglanensis* 172205(T)^T^  (100%)  *Streptomyces fradiae* DSM 40063^T^  (100%)  *Streptomyces paucisporeus* CGMCC 4.2025^T^  (98.93%)  *Sinomonas halotolerans* CFH S0499^T^  (99.73%)  *Streptomyces bingchenggensis* BCW-1^T^  (99.6%)  *Streptomyces luteogriseus* NBRC 13402^T^  (100%)  *Streptomyces tendae* ATCC 19812^T^  (99.87%)  *Streptomyces caeni* HA15955^T^  (99.87%)  *Micromonospora carbonacea* DSM 43168^T^  (100%)  *Sinomonas flava* CW 108^T^  (99.33%)  *Streptomyces anandii* NRRL B-3590^T^  (100%)  *Streptomyces coeruleofuscus* NBRC 12757^T^  (99.33%)  *Micromonospora chersina* DSM 44151^T^  (99.47%)  *Rhodococcus hoagie* DSM 20295^T^  (99.87%)  *Curtobacterium flaccumfaciens* LMG 3645^T^  (99.73%)  *Gordonia terrae* NBRC 100016^T^  (100%)  *Streptomyces viridobrunneus* LMG 20317^T^  (99.87%)  *Micromonospora maritima* D10-9-5^T^  (100%)  *Curtobacterium oceanosedimentum* ATCC 31317^T^  (100%)  *Streptomyces misionensis* DSM 40306^T^  (99.73%)  *Streptomyces cyaneus* NRRL B-2296^T^  (99.33%)  *Curtobacterium citreum* DSM 20528^T^  (99.73%)  *Streptomyces broussonetiae* T44^T^  (99.2%)  *Streptomyces prasinosporus* NRRL B-12431^T^  (98.93%)  *Micromonospora vinacea* GUI63^T^  (99.6%)  *Streptomyces nanshensis* SCSIO 01066^T^  (99.87%)  *Nocardia Testacea* NBRC 100365^T^  (99.87%)  *Micromonospora yasonensis* DS3186^T^  (99.33%)  *Micromonospora chalcea* DSM 43026^T^  (99.87%)  *Actinomadura Mexicana* A290^T^  (99.47%)  *Micromonospora fluminis* A38^T^  (100%)  *Micromonospora haikouensis* 232617^T^  (99.07%)  *Micromonospora saelicesensisLupac* 09^T^  (99.33%)  *Micromonospora rhizosphaerae* DSM 45431^T^  (99.47%)  *Micromonospora chokoriensis* DSM 45160^T^  (100%)  *Mycobacterium grossiae* SCH^T^  (98.3%)  *Mycobacterium wolinskyi* ATCC 700010^T^  (98.58%)  *Gordonia rhizosphere* NBRC 16068^T^  (100%)  *Micromonospora schwarzwaldensis* HKI0641^T^  (99.87%)  *Micromonospora humi* DSM 45647^T^  (99.47%)  *Micromonospora azadirachtae* AZ1-19^T^  (99.87%)  *Streptomyces chartreusis* NBRC 12753^T^  (99.87%)  *Streptomyces griseoincarnatus* LMG 19316^T^  (100%)  *Streptomyces hyaluromycini* NBRC 110483^T^  (99.87%)  *Streptomyces hawaiiensis* NBRC 12784^T^  (99.73%)  *Streptomyces griseoruber* NRRL B-1818^T^  (99.6%)  *Streptomyces cellulosae* NBRC 13027^T^  (100%)  *Streptomyces globosus* LMG 19896^T^  (100%)  *Streptomyces angustmyceticus* NRRL B-2347^T^  (100%)  *Streptomyces albidoflavus* DSM 40455^T^  (99.87%)  *Streptomyces seoulensis* NRRL B-24310^T^  (100%)  *Streptomyces lannensis* TA4-8^T^  (100%)  *Streptomyces reniochalinae* LHW50302^T^  (99.87%)  *Streptomyces corchorusii* DSM 40340^T^  (98.95%)  *Streptomyces olivaceus* NRRL B-3009^T^  (100%)  *Nocardia sienata* IFM 10088^T^  (99.47%)  *Actinomadura geliboluensis* A8036^T^  (100%)  *Streptomyces hygroscopicus* NBRC 13472^T^  (100%)  *Streptomyces cacaoi* NRRL B-1220^T^  (100%)  *Streptomyces qinglanensis* 172205^T^  (98.96%)  *Streptomyces lydicus* ATCC 25470^T^  (99.35%)  *Streptomyces malaysiensis* NBRC 16446^T^  (100%) | E  M  W  E  M  W  E  M  W  E  M  W  E  M  W  E  M  W  E  M  W  E  M  W  E  M  W  E  M  W  E  M  W  E  M  W  E  M  W  E  M  W  E  M  W  E  M  W  E  M  W  E  M  W  E  M  W  E  M  W  E  M  W  E  M  W  E  M  W  E  M  W  E  M  W  E  M  W  E  M  W  E  M  W  E  M  W  E  M  W  E  M  W  E  M  W  E  M  W  E  M  W  E  M  W  E  M  W  E  M  W  E  M  W  E  M  W  E  M  W  E  M  W  E  M  W  E  M  W  E  M  W  E  M  W  E  M  W  E  M  W  E  M  W  E  M  W  E  M  W  E  M  W  E  M  W  E  M  W  E  M  W  E  M  W  E  M  W  E  M  W  E  M  W  E  M  W  E  M  W  E  M  W  E  M  W  E  M  W  E  M  W  E  M  W  E  M  W  E  M  W  E  M  W  E  M  W  E  M  W  E  M  W  E  M  W  E  M  W  E  M  W  E  M  W  E  M  W  E  M  W  E  M  W  E  M  W  E  M  W  E  M  W  E  M  W  E  M  W  E  M  W  E  M  W  E  M  W  E  M | -  -  -  -  -  -  -  -  -  -  -  -  -  -  -  0.00781  0.0313  0.125  -  -  -  -  -  -  -  -  -  -  -  -  -  -  -  0.5  -  -  -  -  -  -  -  -  -  -  -  -  -  -  0.25  -  -  -  -  -  -  -  -  -  -  -  -  -  -  -  -  -  0.0625  -  -  -  -  -  -  -  -  0.0313  -  -  -  -  -  1  -  -  -  -  -  0.25  -  -  0.5  -  -  -  -  -  0.25  0.5  -  1  -  -  0.5  -  -  -  -  -  -  -  -  -  -  -  -  -  -  -  -  -  -  -  -  0.0625  -  -  0.5  -  -  -  -  -  -  -  -  -  -  -  1  -  -  0.00781  0.00781  0.0625  1  -  -  -  -  -  0.25  -  -  -  -  -  -  -  -  -  -  -  -  -  -  0.5  -  -  0.0625  -  -  -  -  -  -  -  -  0.5  -  -  -  -  -  -  -  -  1  -  -  -  -  -  -  -  -  -  -  -  0.5  -  -  0.0625  -  -  -  -  -  0.125  -  -  -  -  -  0.25  -  -  -  -  -  -  -  -  0.25  0.125  -  0.00781  -  -  0.125  -  -  -  -  -  0.5  -  -  -  -  -  -  -  -  0.00781  -  1  0.125  -  -  0.125  1  0.5  0.00781  -  -  -  -  -  0.125  - | -  -  -  -  -  -  -  -  -  -  -  -  -  -  -  0.00781  0.0156  0.0625  -  -  -  -  -  -  -  -  -  -  -  -  -  -  -  0.5  -  -  -  -  -  -  -  -  -  -  -  -  -  -  0.25  -  -  -  -  -  -  -  -  -  -  -  -  -  -  -  -  -  0.0313  -  -  -  -  -  -  -  -  0.0625  -  -  -  -  -  0.5  -  -  -  -  -  0.5  -  -  -  -  -  -  -  -  0.5  0.5  -  -  -  -  0.25  -  -  -  -  -  -  -  -  -  -  -  -  -  -  0.25  -  -  -  -  -  0.0625  0.5  -  0.5  -  -  -  -  -  -  -  -  -  -  -  1  -  -  0.00781  0.00781  0.25  -  -  -  -  -  -  0.25  1  -  -  -  -  -  -  -  -  -  -  -  -  -  0.5  -  -  0.0313  -  -  -  -  -  -  -  -  0.25  -  -  -  -  -  -  -  -  1  -  -  -  -  -  -  -  -  -  -  -  -  -  -  0.125  -  -  -  -  -  -  -  -  -  -  -  0.5  -  -  -  -  -  -  -  -  -  0.0625  -  0.00781  -  -  0.25  -  -  0.5  -  -  0.5  -  -  -  -  -  -  -  -  0.00781  -  1  0.25  0.25  -  0.125  -  0.5  0.00781  -  -  -  -  -  0.25  - | -  -  -  -  -  -  -  -  -  -  -  -  -  -  -  0.125  0.5  -  -  -  -  -  -  -  -  -  -  -  -  -  -  -  -  1  -  -  -  -  -  -  -  -  -  -  -  -  -  -  -  -  0.5  -  -  -  -  -  -  -  -  -  -  -  -  -  -  -  0.25  -  -  -  -  -  -  -  -  -  -  -  -  -  -  -  -  -  -  -  -  -  -  0.5  -  0.0625  -  -  -  -  -  -  -  -  -  -  0.5  -  -  -  -  -  -  -  -  -  -  -  -  -  -  -  -  -  -  -  -  -  0.5  0.5  -  -  -  -  -  -  -  -  -  -  -  -  -  -  -  0.125  0.25  -  -  -  -  -  -  -  -  -  -  -  -  -  -  -  -  -  -  -  -  -  -  -  -  -  -  -  -  -  -  -  -  -  -  -  -  -  -  -  -  -  -  -  -  -  -  -  -  -  -  -  -  -  -  -  -  0.5  -  -  -  -  -  -  -  -  -  -  -  -  -  -  -  -  -  -  -  -  -  -  -  -  -  0.125  -  -  -  -  -  -  -  -  -  1  -  -  -  -  -  -  -  -  -  -  0.5  -  -  -  -  -  -  -  -  -  -  -  -  - | -  -  -  -  -  -  -  -  -  -  -  -  -  -  -  0.0625  -  -  -  -  -  -  -  -  -  -  -  -  -  -  -  -  -  -  -  -  -  -  -  -  -  -  -  -  -  -  -  -  -  -  0.5  -  -  -  -  -  -  -  -  -  -  -  -  -  -  -  0.5  -  -  -  -  -  -  -  -  -  -  -  -  -  -  -  -  -  -  -  -  -  -  0.0156  -  -  -  -  -  -  -  -  -  -  -  -  -  -  -  -  -  -  -  -  -  -  -  -  -  -  -  -  -  -  -  -  -  -  -  -  -  -  -  -  -  -  -  -  -  -  -  -  -  -  -  0.0625  -  -  -  -  -  -  -  -  -  -  -  -  -  -  -  -  -  -  -  -  -  -  -  -  -  -  -  -  -  -  -  -  -  -  -  -  -  -  -  -  -  -  -  -  -  -  -  -  -  -  -  -  -  -  -  -  -  -  -  -  -  -  -  -  -  -  -  -  0.125  -  -  -  -  -  -  -  -  -  -  -  -  -  -  0.125  -  -  -  -  -  -  -  -  -  -  -  -  -  -  -  -  -  -  -  -  0.5  -  -  -  -  -  -  -  -  -  -  -  -  - | -  -  -  -  -  -  -  -  -  -  -  -  -  -  -  0.125  -  -  -  -  -  -  -  -  -  -  -  -  -  -  -  -  -  -  -  -  -  -  -  -  -  -  -  -  -  -  -  -  -  -  -  -  -  -  -  -  -  -  -  -  -  -  -  -  -  -  -  -  -  -  -  -  -  -  -  -  -  -  -  -  -  -  -  -  -  -  -  -  -  -  -  -  -  -  -  -  -  -  -  -  -  -  -  -  -  -  -  -  -  -  -  -  -  -  -  -  -  -  -  -  -  -  -  -  -  -  -  -  -  -  -  -  -  -  -  -  -  -  -  -  -  -  -  -  -  -  -  -  -  -  -  -  -  -  -  -  -  -  -  -  -  -  -  -  -  -  -  -  -  0.5  -  -  -  -  -  -  -  -  -  -  -  -  -  -  -  -  -  -  -  -  -  -  -  -  -  -  -  -  -  -  -  -  -  -  -  -  -  -  -  -  -  -  -  -  -  -  -  -  -  -  -  -  -  -  -  -  -  -  -  -  -  -  -  -  -  -  -  -  -  -  -  -  -  -  -  -  -  -  -  -  -  -  -  0.25  -  -  -  -  -  -  - | -  -  -  -  -  -  -  -  -  -  -  -  -  -  -  0.125  -  -  -  -  -  -  -  -  -  -  -  -  -  -  -  -  -  1  -  -  -  -  -  -  -  -  -  -  -  -  -  -  -  -  -  -  -  -  -  -  -  -  -  -  -  -  -  -  -  -  1  -  -  -  -  -  -  -  -  -  -  -  -  -  -  -  -  -  -  -  -  -  -  -  -  -  -  -  -  -  -  -  -  -  -  -  -  -  -  -  -  -  -  -  -  -  -  -  -  -  -  -  -  -  -  -  -  -  -  -  -  1  -  -  0.5  -  -  1  -  -  -  -  -  -  -  -  -  -  -  -  -  -  -  -  -  -  -  -  -  -  -  -  -  -  -  -  -  -  -  -  1  -  -  -  -  -  -  -  -  -  -  -  -  -  -  -  -  -  -  -  -  -  -  -  -  -  -  -  -  -  -  -  -  -  -  -  -  -  -  -  -  -  -  -  -  -  -  -  -  -  -  -  -  -  -  -  -  -  -  -  -  -  -  -  -  -  -  -  -  0.125  -  -  -  -  -  -  -  -  -  -  -  -  0.125  -  -  1  -  -  -  -  -  -  -  -  - |
|  |  | W | - | - | - | - | - |  |
|  |  |  | 0.00391 | 0.00391 | 0.00391 | 0.00391 | 0.00391 | 0.00195 |

Note: ^a^ E: crude sample extracted with ethyl acetate; M: crude sample from mycelium; W: crude sample from water layer;

^b^ The unit of minimum inhibitory concentration (MIC): mg/mL; —, no inhibition;

positive control: Ciprofloxacin and amphotericin B；

(S)：Sensitive strain ; (R)：Drug-resistant strain.

**Table S4 Immunosuppressive activity of culturable Actinobacteria from mangrove soil and and their similarity of 16S rRNA gene sequences**

| Isolate(GenBank accession no.) | Genus and species (Similanity,%) | inhibition rate %（±） | | |
| --- | --- | --- | --- | --- |
|  |  | **E** | M | W |
| A-2  (OM019219)  A-4  (OM019241)  A-12  (OM019213)  A-23  (OM019222)  A-29  (OM019229)  A-30  (OM019232)  A-33  (OM019236)  A-41  (OM019242)  A-43  (OM019244)  A-49  (OM019245)  A-54  (OM019251)  A-56  (OM019252)  A-57  (OM019254)  A-62  (OM019257)  A-68  (OM019260)  A-69  (OM019259)  A-70  (OM019261)  A-72  (OM019262)  A-74  (OM019263)  A-90  (OM019264)  A-92  (OM019265)  A-103  (OM019210)  A-107  (OM019211)  A-116  (OM019212)  A-117  (OM019294)  A-119  (OM019295)  A-123  (OM019266)  A-124  (OM019214)  A-136  (OM019215)  A-189  (OM019296)  A-194  (OM019216)  A-196  (OM019217)  A-198  (OM019218)  A-217  (OM019220)  A-229  (OM019221)  A-230  (OM019223)  A-244  (OM019224)  A-251  (OM019225)  A-252  (OM019226)  A-259  (OM019227)  A-260  (OM019267)  A-261  (OM019268)  A-272  (OM019228)  A-290  (OM019230)  A-299  (OM019231)  A-318  (OM019233)  A-320  (OM019234)  A-326  (OM019235)  A-369  (OM019237)  A-379  (OM019269)  A-380  (OM019238)  A-383  (OM019239)  A-399  (OM019240)  A-426  (OM019243)  A-493  (OM019246)  A-496  (OM019247)  A-498  (OM019248)  A-529  (OM019249)  A-537  (OM019250)  A-563  (OM019270)  A-568  (OM019253)  A-569  (OM019271)  A-570  (OM019255)  A-584  (OM019256)  A-635  (OM019272)  A-638  (OM019258)  J-12  (OM019273)  J-14  (OM019274)  J-26  (OM019280)  J-31  (OM019281)  J-33  (OM019293)  J-66  (OM019282)  J-68  (OM019283)  J-71  (OM019284)  J-140  (OM019275)  J-151  (OM019276)  J-155  (OM019277)  J-167  (OM019278)  J-170  (OM019279)  R-8  (OM019287)  R-55  (OM019285)  R-77  (OM019286)  ZRJ-1  (OM019288)  ZRJ-2  (OM019289)  ZRJ-11  (OM019291)  ZRJ-12  (OM019292)  ZRJ-20  (OM019290) | *Streptomyces venetus* CMU-AB225^T^  (99.6%)  *Curtobacterium luteum* DSM 20542^T^  (99.87%)  *Streptomyces aurantiogriseus* NBRC 12842^T^  (99.2%)  *Nocardia arthritidis* NBRC 100137^T^  (99.07%)  *Streptomyces sanyensis* 219820^T^  (100%)  *Streptomyces parvulus* NBRC 13193^T^  (100%)  *Streptomyces chromofuscus* NBRC 12851^T^  (98.8%)  *Streptomyces glauciniger* CGMCC 4.1858^T^  (99.47%)  *Streptomyces bungoensis* DSM 41781^T^  (99.87%)  *Streptomyces sundarbansensis* MS1/7^T^  (99.73%)  *Streptomyces daghestanicus* NRRL B-5418^T^  (100%)  *Streptomyces pseudogriseolus* NRRL B-3288^T^  (100%)  *Streptomyces carpinensis* NRRL B-16921^T^  (98.54%)  *Streptomyces leeuwenhoekii* C34^T^  (98.55%)  *Streptomyces qinglanensis* 172205^T^  (99.87%)  *Streptomyces drozdowiczii* NBRC 101007^T^  (99.47)  *Streptomyces caniferus* NBRC 15389^T^  (100%)  *Streptomyces nogalater* JCM 4799^T^  (98.68%)  *Streptomyces lydicus* ATCC 25470^T^  (99.73%)  *Streptomyces pluripotens* MUSC 135^T^  (99.87%)  *Streptomyces aldersoniae* NRRL 18513^T^  (99.6%)  *Microbacterium paraoxydans* NBRC 103076^T^  (99.6%)  *Streptomyces racemochromogenes* NRRL B-5430^T^  (99.6%)  *Streptomyces badius* NRRL B-2567^T^  (99.73%)  *Streptomyces malaysiense* MUSC 136^T^  (100%)  *Streptomyces qinglanensis* 172205(T)^T^  (100%)  *Streptomyces fradiae* DSM 40063^T^  (100%)  *Streptomyces paucisporeus* CGMCC 4.2025^T^  (98.93%)  *Sinomonas halotolerans* CFH S0499^T^  (99.73%)  *Streptomyces bingchenggensis* BCW-1^T^  (99.6%)  *Streptomyces luteogriseus* NBRC 13402^T^  (100%)  *Streptomyces tendae* ATCC 19812^T^  (99.87%)  *Streptomyces caeni* HA15955^T^  (99.87%)  *Micromonospora carbonacea* DSM 43168^T^  (100%)  *Sinomonas flava* CW 108^T^  (99.33%)  *Streptomyces anandii* NRRL B-3590^T^  (100%)  *Streptomyces coeruleofuscus* NBRC 12757^T^  (99.33%)  *Micromonospora chersina* DSM 44151^T^  (99.47%)  *Rhodococcus hoagie* DSM 20295^T^  (99.87%)  *Curtobacterium flaccumfaciens* LMG 3645^T^  (99.73%)  *Gordonia terrae* NBRC 100016^T^  (100%)  *Streptomyces viridobrunneus* LMG 20317^T^  (99.87%)  *Micromonospora maritima* D10-9-5^T^  (100%)  *Curtobacterium oceanosedimentum* ATCC 31317^T^  (100%)  *Streptomyces misionensis* DSM 40306^T^  (99.73%)  *Streptomyces cyaneus* NRRL B-2296^T^  (99.33%)  *Curtobacterium citreum* DSM 20528^T^  (99.73%)  *Streptomyces broussonetiae* T44^T^  (99.2%)  *Streptomyces prasinosporus* NRRL B-12431^T^  (98.93%)  *Micromonospora vinacea* GUI63^T^  (99.6%)  *Streptomyces nanshensis* SCSIO 01066^T^  (99.87%)  *Nocardia Testacea* NBRC 100365^T^  (99.87%)  *Micromonospora yasonensis* DS3186^T^  (99.33%)  *Micromonospora chalcea* DSM 43026^T^  (99.87%)  *Actinomadura Mexicana* A290^T^  (99.47%)  *Micromonospora fluminis* A38^T^  (100%)  *Micromonospora haikouensis* 232617^T^  (99.07%)  *Micromonospora saelicesensisLupac* 09^T^  (99.33%)  *Micromonospora rhizosphaerae* DSM 45431^T^  (99.47%)  *Micromonospora chokoriensis* DSM 45160^T^  (100%)  *Mycobacterium grossiae* SCH^T^  (98.3%)  *Mycobacterium wolinskyi* ATCC 700010^T^  (98.58%)  *Gordonia rhizosphere* NBRC 16068^T^  (100%)  *Micromonospora schwarzwaldensis* HKI0641^T^  (99.87%)  *Micromonospora humi* DSM 45647^T^  (99.47%)  *Micromonospora azadirachtae* AZ1-19^T^  (99.87%)  *Streptomyces chartreusis* NBRC 12753^T^  (99.87%)  *Streptomyces griseoincarnatus* LMG 19316^T^  (100%)  *Streptomyces hyaluromycini* NBRC 110483^T^  (99.87%)  *Streptomyces hawaiiensis* NBRC 12784^T^  (99.73%)  *Streptomyces griseoruber* NRRL B-1818^T^  (99.6%)  *Streptomyces cellulosae* NBRC 13027^T^  (100%)  *Streptomyces globosus* LMG 19896^T^  (100%)  *Streptomyces angustmyceticus* NRRL B-2347^T^  (100%)  *Streptomyces albidoflavus* DSM 40455^T^  (99.87%)  *Streptomyces seoulensis* NRRL B-24310^T^  (100%)  *Streptomyces lannensis* TA4-8^T^  (100%)  *Streptomyces reniochalinae* LHW50302^T^  (99.87%)  *Streptomyces corchorusii* DSM 40340^T^  (98.95%)  *Streptomyces olivaceus* NRRL B-3009^T^  (100%)  *Nocardia sienata* IFM 10088^T^  (99.47%)  *Actinomadura geliboluensis* A8036^T^  (100%)  *Streptomyces hygroscopicus* NBRC 13472^T^  (100%)  *Streptomyces cacaoi* NRRL B-1220^T^  (100%)  *Streptomyces qinglanensis* 172205^T^  (98.96%)  *Streptomyces lydicus* ATCC 25470^T^  (99.35%)  *Streptomyces malaysiensis* NBRC 16446^T^  (100%) | 32.2±0.86  57.3±1.37  34.0±1.61  63.75±0.9  27.3±0.86  33.1±1.37  33.3±2.23  52.92±2.7  65.2±1.04  49.0±0.71  42.5±1.48  51.5±0.71  65.42±1.8  49.7±2.27  52.7±0.86  55.2±0.52  56.6±2.04  61.1±2.55  52.27±0.5  45.2±1.99  54.9±1.64  49.3±0.19  54.1±1.64  42.27±1.8  53.4±1.05  40.8±0.98  42.5±1.54  59.4±1.32  56.4±1.18  53.6±1.31  60.1±1.05  42.2±1.15  51.07±0.5  48.4±2.47  41.7±1.61  61.2±1.18  49.0±1.12  55.8±0.43  57.8±1.5  61.7±0.94  54.7±1.32  64.2±1.35  69.5±3.38  46.0±1.89  44.1±2.13  41.5±2.19  55.8±3.27  56.1±2.44  44.1±1.51  58.4±2.44  51.9±1.51  48.3±2.13  51.5±1.69  48.4±1.92  57.4±2.19  48.4±1.77  50.1±1.56  51.53±1.2  54.9±2.21  30.6±3.79  43.9±1.92  30.1±2.07  76.84±1.6  39.0±7.94  40.69±1.1  39.94±0.7  42.3±1.25  53.3±0.55  59.9±0.67  42.8±1.49  41.5±1.49  41.3±0.75  47.5±0.44  65.0±0.86  49.1±0.89  57.55±1.1  47.3±2.02  75.8±1.72  43.0±0.89  40.1±0.54  44.7±0.44  57.5±1.29  54.34±1  63.3±0.54  51.5±1.06  59.9±1.13  60.8±0.65 | 6.18±1.66  9.68±2.37  22.85±5.36  44.09±0.38  45.7±1.01  12.63±0.38  23.12±1.01  30.11±2.31  43.82±1.9  31.72±1.37  7.8±1.66  18.01±2.74  26.34±1.52  50±4.75  53.76±4.28  29.57±1.66  8.6±1.66  16.67±2.49  33.89±2.97  34.03±1.04  33.33±5.01  37.5±0.9  31.81±1.87  38.19±1.09  51.39±1.87  41.39±0.52  41.53±1.29  34.58±1.23  44.03±0.71  28.06±2.41  40.42±1.23  39.31±0.71  40.69±0.2  48.06±3.44  40.28±1.29  38.19±2.75  24.31±1.19  33.33±1.7  17.5±8.56  24.72±0.86  29.03±0.39  31.67±1.36  50.69±0.79  32.08±1.48  26.94±0.71  35±1.89  41.25±0.9  38.75±1.56  33.61±0.52  38.89±0.86  43.47±1.19  41.11±0.52  31.94±1.71  30.14±1.37  34.44±1.29  38.47±1.37  35.69±1.37  32.36±0.71  42.92±1.23  48.89±2.08  9.72±1.19  32.36±1.37  33.89±2.05  39.72±2.51  37.5±2.38  35.14±3.49  45.42±0.59  12.36±1.87  46.11±3.91  41.86±1.9  42.33±0.38  39.22±1.44  15.19±0.96  48.37±1.74  42.33±1.74  39.22±0.58  41.55±0.96  43.88±0.22  48.68±2.95  44.19±2.66  38.91±2.53  39.07±2.01  38.6±1.14  44.5±1.16  31.78±2.23  32.09±0.38  46.51±3.11 | 26.67±1.24  17.45±0.59  7.76±1.24  10.55±3.27  12.61±2.4  22.42±2.09  4.61±2.25  14.79±4.16  17.94±1.74  10.18±0.3  7.15±0.91  16.36±1.81  8±2.14  12.61±2.69  17.33±1.2  7.39±1.91  8.48±0.17  20.24±2.25  6.59±0.85  14.57±5.02  19.56±0.56  25.15±4.4  20.56±2.78  29.54±1.02  31.34±4.38  16.77±2.24  10.58±2.78  11.98±2.59  21.56±4.4  23.55±2.2  10.78±3.42  16.57±6.29  18.56±0.98  16.77±4.48  25.35±2.31  26.55±1.72  9.91±4.14  5.86±1.15  9.23±4.94  7.21±2.23  10.81±0.55  11.71±1.15  6.08±3.07  7.43±3.36  5.86±1.94  13.29±0.84  10.81±1.66  7.66±1.15  15.32±2.09  21.4±1.15  6.08±0.55  25.23±4.69  9.68±1.15  6.76±1.46  2.48±1.27  14.41±0.64  7.88±2.09  6.31±2.3  24.63±0.84  16.46±1.35  14.15±2.46  10.48±1.84  14.69±1.2  14.15±1.84  20.95±2.22  12.24±4.37  18.91±1.5  23.67±2  29.52±0.69  32.52±0.51  11.84±0.67  9.93±8.83  16.6±0.19  21.09±2.87  22.86±0.33  2.31±1.02  4.76±1.17  3.67±1.73  11.29±0.51  19.32±1.02  17.41±1.64  15.78±1.9  33.88±0.67  13.74±0.84  41.77±0.51  31.7±1.68  13.74±1.71 |

Note：E: crude sample extracted with ethyl acetate; M: crude sample from mycelium; W: crude sample from water layer.

**Table S5 Anticancer activity of culturable Actinobacteria from mangrove soil and their similarity of 16S rRNA gene sequences**

| Isolate(GenBank accession no.) | Genus and species (Similanity,%) | Fractions^a^ | inhibition rate %（±） | | |
| --- | --- | --- | --- | --- | --- |
|  |  |  | HepG2 | Hela | HCT-116 |
| A-2  (OM019219)  A-4  (OM019241)  A-12  (OM019213)  A-23  (OM019222)  A-29  (OM019229)  A-30  (OM019232)  A-33  (OM019236)  A-41  (OM019242)  A-43  (OM019244)  A-49  (OM019245)  A-54  (OM019251)  A-56  (OM019252)  A-57  (OM019254)  A-62  (OM019257)  A-68  (OM019260)  A-69  (OM019259)  A-70  (OM019261)  A-72  (OM019262)  A-74  (OM019263)  A-90  (OM019264)  A-92  (OM019265)  A-103  (OM019210)  A-107  (OM019211)  A-116  (OM019212)  A-117  (OM019294)  A-119  (OM019295)  A-123  (OM019266)  A-124  (OM019214)  A-136  (OM019215)  A-189  (OM019296)  A-194  (OM019216)  A-196  (OM019217)  A-198  (OM019218)  A-217  (OM019220)  A-229  (OM019221)  A-230  (OM019223)  A-244  (OM019224)  A-251  (OM019225)  A-252  (OM019226)  A-259  (OM019227)  A-260  (OM019267)  A-261  (OM019268)  A-272  (OM019228)  A-290  (OM019230)  A-299  (OM019231)  A-318  (OM019233)  A-320  (OM019234)  A-326  (OM019235)  A-369  (OM019237)  A-379  (OM019269)  A-380  (OM019238)  A-383  (OM019239)  A-399  (OM019240)  A-426  (OM019243)  A-493  (OM019246)  A-496  (OM019247)  A-498  (OM019248)  A-529  (OM019249)  A-537  (OM019250)  A-563  (OM019270)  A-568  (OM019253)  A-569  (OM019271)  A-570  (OM019255)  A-584  (OM019256)  A-635  (OM019272)  A-638  (OM019258)  J-12  (OM019273)  J-14  (OM019274)  J-26  (OM019280)  J-31  (OM019281)  J-33  (OM019293)  J-66  (OM019282)  J-68  (OM019283)  J-71  (OM019284)  J-140  (OM019275)  J-151  (OM019276)  J-155  (OM019277)  J-167  (OM019278)  J-170  (OM019279)  R-8  (OM019287)  R-55  (OM019285)  R-77  (OM019286)  ZRJ-1  (OM019288)  ZRJ-2  (OM019289)  ZRJ-11  (OM019291)  ZRJ-12  (OM019292)  ZRJ-20  (OM019290) | *Streptomyces venetus* CMU-AB225^T^  (99.6%)  *Curtobacterium luteum* DSM 20542^T^  (99.87%)  *Streptomyces aurantiogriseus* NBRC 12842^T^  (99.2%)  *Nocardia arthritidis* NBRC 100137^T^  (99.07%)  *Streptomyces sanyensis* 219820^T^  (100%)  *Streptomyces parvulus* NBRC 13193^T^  (100%)  *Streptomyces chromofuscus* NBRC 12851^T^  (98.8%)  *Streptomyces glauciniger* CGMCC 4.1858^T^  (99.47%)  *Streptomyces bungoensis* DSM 41781^T^  (99.87%)  *Streptomyces sundarbansensis* MS1/7^T^  (99.73%)  *Streptomyces daghestanicus* NRRL B-5418^T^  (100%)  *Streptomyces pseudogriseolus* NRRL B-3288^T^  (100%)  *Streptomyces carpinensis* NRRL B-16921^T^  (98.54%)  *Streptomyces leeuwenhoekii* C34^T^  (98.55%)  *Streptomyces qinglanensis* 172205^T^  (99.87%)  *Streptomyces drozdowiczii* NBRC 101007^T^  (99.47)  *Streptomyces caniferus* NBRC 15389^T^  (100%)  *Streptomyces nogalater* JCM 4799^T^  (98.68%)  *Streptomyces lydicus* ATCC 25470^T^  (99.73%)  *Streptomyces pluripotens* MUSC 135^T^  (99.87%)  *Streptomyces aldersoniae* NRRL 18513^T^  (99.6%)  *Microbacterium paraoxydans* NBRC 103076^T^  (99.6%)  *Streptomyces racemochromogenes* NRRL B-5430^T^  (99.6%)  *Streptomyces badius* NRRL B-2567^T^  (99.73%)  *Streptomyces malaysiense* MUSC 136^T^  (100%)  *Streptomyces qinglanensis* 172205(T)^T^  (100%)  *Streptomyces fradiae* DSM 40063^T^  (100%)  *Streptomyces paucisporeus* CGMCC 4.2025^T^  (98.93%)  *Sinomonas halotolerans* CFH S0499^T^  (99.73%)  *Streptomyces bingchenggensis* BCW-1^T^  (99.6%)  *Streptomyces luteogriseus* NBRC 13402^T^  (100%)  *Streptomyces tendae* ATCC 19812^T^  (99.87%)  *Streptomyces caeni* HA15955^T^  (99.87%)  *Micromonospora carbonacea* DSM 43168^T^  (100%)  *Sinomonas flava* CW 108^T^  (99.33%)  *Streptomyces anandii* NRRL B-3590^T^  (100%)  *Streptomyces coeruleofuscus* NBRC 12757^T^  (99.33%)  *Micromonospora chersina* DSM 44151^T^  (99.47%)  *Rhodococcus hoagie* DSM 20295^T^  (99.87%)  *Curtobacterium flaccumfaciens* LMG 3645^T^  (99.73%)  *Gordonia terrae* NBRC 100016^T^  (100%)  *Streptomyces viridobrunneus* LMG 20317^T^  (99.87%)  *Micromonospora maritima* D10-9-5^T^  (100%)  *Curtobacterium oceanosedimentum* ATCC 31317^T^  (100%)  *Streptomyces misionensis* DSM 40306^T^  (99.73%)  *Streptomyces cyaneus* NRRL B-2296^T^  (99.33%)  *Curtobacterium citreum* DSM 20528^T^  (99.73%)  *Streptomyces broussonetiae* T44^T^  (99.2%)  *Streptomyces prasinosporus* NRRL B-12431^T^  (98.93%)  *Micromonospora vinacea* GUI63^T^  (99.6%)  *Streptomyces nanshensis* SCSIO 01066^T^  (99.87%)  *Nocardia Testacea* NBRC 100365^T^  (99.87%)  *Micromonospora yasonensis* DS3186^T^  (99.33%)  *Micromonospora chalcea* DSM 43026^T^  (99.87%)  *Actinomadura Mexicana* A290^T^  (99.47%)  *Micromonospora fluminis* A38^T^  (100%)  *Micromonospora haikouensis* 232617^T^  (99.07%)  *Micromonospora saelicesensisLupac* 09^T^  (99.33%)  *Micromonospora rhizosphaerae* DSM 45431^T^  (99.47%)  *Micromonospora chokoriensis* DSM 45160^T^  (100%)  *Mycobacterium grossiae* SCH^T^  (98.3%)  *Mycobacterium wolinskyi* ATCC 700010^T^  (98.58%)  *Gordonia rhizosphere* NBRC 16068^T^  (100%)  *Micromonospora schwarzwaldensis* HKI0641^T^  (99.87%)  *Micromonospora humi* DSM 45647^T^  (99.47%)  *Micromonospora azadirachtae* AZ1-19^T^  (99.87%)  *Streptomyces chartreusis* NBRC 12753^T^  (99.87%)  *Streptomyces griseoincarnatus* LMG 19316^T^  (100%)  *Streptomyces hyaluromycini* NBRC 110483^T^  (99.87%)  *Streptomyces hawaiiensis* NBRC 12784^T^  (99.73%)  *Streptomyces griseoruber* NRRL B-1818^T^  (99.6%)  *Streptomyces cellulosae* NBRC 13027^T^  (100%)  *Streptomyces globosus* LMG 19896^T^  (100%)  *Streptomyces angustmyceticus* NRRL B-2347^T^  (100%)  *Streptomyces albidoflavus* DSM 40455^T^  (99.87%)  *Streptomyces seoulensis* NRRL B-24310^T^  (100%)  *Streptomyces lannensis* TA4-8^T^  (100%)  *Streptomyces reniochalinae* LHW50302^T^  (99.87%)  *Streptomyces corchorusii* DSM 40340^T^  (98.95%)  *Streptomyces olivaceus* NRRL B-3009^T^  (100%)  *Nocardia sienata* IFM 10088^T^  (99.47%)  *Actinomadura geliboluensis* A8036^T^  (100%)  *Streptomyces hygroscopicus* NBRC 13472^T^  (100%)  *Streptomyces cacaoi* NRRL B-1220^T^  (100%)  *Streptomyces qinglanensis* 172205^T^  (98.96%)  *Streptomyces lydicus* ATCC 25470^T^  (99.35%)  *Streptomyces malaysiensis* NBRC 16446^T^  (100%) | E  M  W  E  M  W  E  M  W  E  M  W  E  M  W  E  M  W  E  M  W  E  M  W  E  M  W  E  M  W  E  M  W  E  M  W  E  M  W  E  M  W  E  M  W  E  M  W  E  M  W  E  M  W  E  M  W  E  M  W  E  M  W  E  M  W  E  M  W  E  M  W  E  M  W  E  M  W  E  M  W  E  M  W  E  M  W  E  M  W  E  M  W  E  M  W  E  M  W  E  M  W  E  M  W  E  M  W  E  M  W  E  M  W  E  M  W  E  M  W  E  M  W  E  M  W  E  M  W  E  M  W  E  M  W  E  M  W  E  M  W  E  M  W  E  M  W  E  M  W  E  M  W  E  M  W  E  M  W  E  M  W  E  M  W  E  M  W  E  M  W  E  M  W  E  M  W  E  M  W  E  M  W  E  M  W  E  M  W  E  M  W  E  M  W  E  M  W  E  M  W  E  M  W  E  M  W  E  M  W  E  M  W  E  M  W  E  M  W  E  M  W  E  M  W  E  M  W  E  M  W  E  M  W  E  M  W  E  M  W  E  M  W  E  M  W  E  M  W  E  M  W  E  M  W  E  M  W  E  M  W | 62.21±4.01  26.88±0.41  9.47±5.46  42.82±0.72  24.29±5.75  25.16±18.52  69.75±2.39  23.87±12.82  28.94±7.87  24.57±1.63  16.08±5.85  30.65±13.04  81.1±0.23  2.64±0.72  11.91±6.36  64.96±4.78  32.01±3.1  6.06±3.82  47.45±14.32  23.87±11.18  14.07±9.86  75.1±0.7  3.02±1.23  32.11±7.57  45.34±7.36  20.6±1.79  12.28±9.46  75.83±0.94  9.63±3.29  7.32±4.45  38.77±2.19  13.32±3.8  4.51±2.64  13.54±1.69  11.81±9.38  16.02±8.67  61.07±4.87  18.93±0.72  2.56±1.56  45.74±12.32  6.45±1.65  9.02±5.73  58.8±14.68  13.32±8.3  10.57±3.44  67.96±2.39  30.62±9.86  32.68±5.53  61.8±8.29  3.43±0.93  51.3±25.39  49.07±0.64  7.87±4.78  20.04±12.37  49.96±4.14  14.07±6.98  9.88±6.75  49.23±0.8  26.8±15.82  11.26±5.97  56.77±0.61  16.83±0.21  14.72±8.61  78.67±2.2  11.31±2.62  25.45±1.64  73.72±1.82  1.51±0.82  9.55±3.39  63.99±3.79  9.63±3.9  15.08±11.57  68.29±7.46  3.67±2.71  16.38±0.35  81.91±0.23  12.03±1.11  22.4±14.57  44.69±0.9  2.6±1.65  36.63±1.74  57.66±2.69  15.75±5.23  24.59±11.35  52.72±12.12  7.2±3.59  3.82±2.09  57.58±5.37  19.26±3.08  21.26±0.76  69.83±4.98  7.12±1.33  8.54±6.82  70.8±2.02  22.03±1.74  5.16±2.82  78.67±0.75  28.89±0.54  3.29±0.62  77.78±3.02  11.98±6.67  16.75±11.58  69.18±7.67  29.4±8.52  18.54±12  76.24±1.16  20.27±12.25  40.41±0.3  68.53±4.77  21.52±12.93  11.91±6.83  75.51±1.21  4.19±1.03  12.56±6.98  71.29±3.39  12.4±6.69  13.82±7.94  72.67±4.37  4.19±0.52  18.58±10.19  77.05±0.7  40.28±1.65  14.63±10.16  72.83±2.11  7.79±5.82  17.6±9.06  44.44±9.05  15.66±12.1  8.41±5.58  76.8±0.9  5.7±2.87  20.85±1.25  73.64±3.39  13.15±3.28  43.54±11.35  74.83±2.2  15.33±10.13  79.85±4.03  75.21±2.56  42.8±27.08  56.3±2.67  74.18±2.71  71.44±0.43  63.38±5.56  69.75±4.41  21.85±15.33  59.85±5.46  76.72±1.4  47.23±9.06  53.19±25.53  77.6±1.59  19.92±0.51  38.25±12.59  76.24±2.08  43.22±1.87  47.8±9.95  70.13±2.92  25.03±4.74  60.15±4.92  75.28±1.52  37.07±1.46  52.37±12.91  40.32±4.99  27.46±9.23  35.78±5.13  72.08±1.68  19.64±2.37  27.02±3.85  76.89±1.82  54.56±3.14  32.43±12.98  77.59±1.41  54.56±0.17  39.88±0.57  29.66±4.33  45.37±9.83  17.41±12.3  56.07±3.74  30.77±0.35  33.77±2.32  67.36±4.7  38.17±4.49  36.54±8.34  72.26±5.35  31.6±5.84  46.55±0.12  76.8±1.95  31.4±6.01  8.68±3.21  70.65±4.48  34.58±1.02  34.85±17.11  71.1±1.68  38.52±0.96  38.25±13.23  76.71±1.91  26.69±7.46  76.8±0.61  70.3±1.79  5.26±3.18  28.33±12.76  74.73±2.66  46.89±2.37  19.1±13.14  75.09±1.65  37.48±3.64  35.75±12.87  70.03±2.3  27.8±1.19  40.82±31.59  66.19±2.82  20.06±4.93  17.47±3.82  45.65±13.21  35.82±4.91  13.33±5.07  32.72±4.39  45.64±5.26  22.97±11.52  73.13±2.9  46.82±2.16  55.78±2.68  41.56±3.95  36.17±10.25  14.99±10.31  73.67±3.06  17.29±0.76  49.49±9.95  51.72±3.85  14.73±7.37  8.12±2.03  52.58±9.34  25.38±13.22  38.89±9.38  57.5±6.22  14.87±10.16  49.02±8.77  71.52±2.08  51.45±0.94  25.62±7.38  71.28±1.76  35.82±8.3  36.19±19.21  78.31±2.59  47.86±4.32  9.96±5.64  41.31±4.95  39.83±14.48  35.08±16.05  24.32±9.85  24.9±13.65  48.82±19.75  60.61±3.72  17.57±12.28  5.56±0.25  53.49±4.06  22.48±5.76  11.79±6.73  69.29±4.68  35.06±16.6  46.03±1.72 | 56.89±3.9  10.71±3.58  25.78±0.77  65.52±3.35  14.05±11.16  49.17±7.22  54.6±3.97  7.12±3.47  62.39±5.64  59.87±2.33  18.43±11.85  22.17±4.78  47.05±1.73  9.37±3.01  75.03±0.95  58.29±2.1  18.12±14.39  20.74±3.52  64.51±2.34  29.93±2.67  69.61±1.35  53.9±0.82  30.58±0.54  54.51±3.93  47.87±0.5  18.84±2.12  47.92±3.7  41.14±3.35  12.17±6.81  32.53±2.09  60.38±1.73  12.9±5.5  58.32±0.73  59.56±0.77  16.3±1.69  45.45±0.5  59.75±1.65  9.42±7  55.69±1.69  56.76±1.73  4.06±2.08  79.89±0.67  58.86±1.48  8.12±5.96  64.15±3.96  51.81±2.91  27.75±3.99  80.7±0.73  55.37±1.79  19.78±2.76  59.78±1.58  51.24±3.27  10.07±5.93  51.47±1.65  46.67±4.2  10.29±6.66  51.44±8.43  52.19±1.56  12.1±4.57  30.97±5.68  61.02±4.76  12.75±6.62  8.59±5.82  63.24±2.1  9.86±3.9  61.31±4.07  55.62±2.64  12.75±6.51  78.28±0.07  58.67±1.87  20.51±5.28  50.75±1.69  29.21±20.84  12.61±6.4  79.38±1.65  43.49±1.26  21.3±2.76  55.48±5.28  40.06±2.26  14.35±8.19  56.67±4.36  50.48±1.09  5.65±2.91  42.46±3.89  45.21±0.39  13.04±6.45  82.02±1.36  43.81±3.89  24.71±2.49  52.3±12.43  58.16±0.24  12.83±6.11  67.21±2.97  45.65±1.09  22.46±5.42  63.38±4.4  43.94±1.48  16.67±10.44  20.92±4.11  56.95±6.22  12.1±3.57  26.42±11.12  54.86±2.18  3.84±0.41  60.59±1.58  45.78±5.29  27.97±0.74  54.67±4.88  22.1±4.28  5.29±2.32  60.8±1.92  45.14±7.63  13.84±9.72  82.35±0.38  50.35±6.07  20.29±4.9  49.34±8.5  45.52±1.79  29.86±8.08  45.75±2.26  52.25±1.56  11.96±2.02  50.36±2.39  58.67±4.28  16.81±11.54  59.04±0.78  54.73±2.43  8.19±1.65  73.7±7.41  43.94±3.82  8.33±6.06  26.54±1.65  36.89±0.18  14.57±7.91  55.8±2.21  54.6±5.06  24.06±1.69  46.83±0.49  42.34±2.31  2.32±1.79  60.26±0.78  53.95±2.17  12.1±7.92  84.62±0.22  47.69±3.16  19.13±2.76  80.64±0.11  38.81±2.39  13.41±10.39  81.81±1.51  46.78±6.63  21.14±4.69  80.25±1.36  37.47±3.01  34.73±3.61  82.38±1.65  45.68±1.01  16.32±1.82  77.23±3.01  46.05±6.18  42.32±4.69  75.73±2.46  26.34±3.81  17.9±4.14  25.4±1.77  36.44±0.85  8.51±3.25  4.83±2.24  41.85±1.5  13.44±10.15  22.89±0.62  30.11±3.73  27.26±7.53  28.93±15.49  38.87±2.31  18.82±0.41  28.73±6.73  26.64±4.15  10.9±0.82  42.92±2.22  32±1.2  25.49±1.26  11.89±6.34  57.18±0.91  14.99±3.43  38.35±3.99  39.42±2.19  56.32±4.44  52.06±5.86  32.3±1.27  12.71±0.63  2.63±1.71  24.03±4.42  13.63±9.3  2.02±1.51  47.81±3.5  30.13±5.51  7.4±4.57  47.45±4.92  4.16±3.07  35.5±9.4  36.01±1.49  14.4±5.82  11.84±6.97  51.52±6.93  15.29±4.37  5.15±0.22  53.95±4.32  20.81±2.81  29.66±6.59  46.23±3.2  42.28±4.6  2.7±0.68  48.36±1.34  17.9±5.51  14.26±4.91  43.92±4.77  25.6±1.88  2.47±0.79  53.28±0.15  28.91±10.06  11.71±6.37  28.71±5.02  31.12±8.08  8.76±0.08  54.01±0.68  22.14±7.49  3.09±2.36  36.01±6.71  27.48±0.6  5.52±3.39  36.5±10.48  31.57±2.71  50.24±3.09  48.11±3.66  17.64±0.91  33.99±7.04  33.64±2.62  15.62±6.76  18.32±3.51  43.37±4.25  37.46±1  14.1±10  63.38±0.6  18.56±5.64  25.84±10.89  47.14±5.29  30.46±3.18  28.69±10.3  32.73±4.69  17.13±13.65  10.72±5.73  39.96±8.57  14.25±1.52  2.63±0.69  24.27±11.57  10.24±5.85  2.52±2.26  43.8±4.33  36.32±5.62  10.22±6.7 | 69.25±2.75  7.77±3.12  36.57±7.24  36.63±0.81  12.68±8.97  26.09±7.5  16.5±11.47  15.34±0.3  2.67±1.79  56.11±1.63  59.87±0.51  1.69±1.08  58.42±2.65  15.13±6.78  12.8±6.77  61.55±0.62  5.6±4.29  2.25±0.98  63.37±1.21  16.18±4.23  2.95±1.72  24.26±1.07  7.7±1.55  3.31±1.86  24.59±6.69  6.02±2.88  19.48±4.57  8.91±3.31  17.44±11.79  11.04±0.7  61.72±0.84  15.13±10.19  6.82±0.95  61.22±3.24  7.42±5.12  15.12±10.85  52.48±12.33  15.2±8.79  5.13±2.69  11.55±7.69  10.64±0.99  4.57±0.78  58.25±6  3.64±2.43  2.95±3.13  45.87±6.47  31.86±3.78  8.79±5.27  38.94±7.9  19.89±10.47  34.53±0.43  10.56±2.47  33.4±18.59  7.31±5.32  2.31±0.93  8.05±5.06  4.5±2.93  38.45±12.13  25.7±20.67  12.87±8.66  22.94±4.07  10.36±5.58  27.43±2.5  18.32±13.98  26.96±3.57  12.59±7.41  20.3±0.4  13.24±0.3  6.12±3.65  43.4±4.68  13.03±7.13  5.77±3.63  58.09±7.07  22.27±17.35  20.18±8.01  47.19±3.76  11.06±7.55  14.84±3.28  25.08±5.09  13.1±9.02  6.19±1.15  44.55±14.76  18.98±11.39  11.32±4.39  54.46±10.11  9.1±6.03  20.89±7.92  66.17±1.42  27.38±3.73  24.26±3.36  42.57±12.37  22.06±0.69  65.33±1.04  15.18±9.78  13.45±9.51  22.71±2.5  69.31±2.02  8.61±6.96  1.48±0.79  33.99±7.1  17.44±1.81  7.45±4.21  21.95±4.05  6.93±3.96  1.13±0.55  30.53±8.31  9.73±4.55  11.18±6.54  27.23±1.85  41.67±27.19  5.7±3.39  29.21±2.91  15.76±10.42  20.6±12.44  23.27±5.87  3.99±2.53  13.08±1.72  64.03±2.63  6.86±4.74  16.17±8.79  31.02±1.02  53.78±14.58  34.95±1.66  30.36±5.28  24.72±7.98  13.85±8.21  24.75±13.41  22.69±16.43  2.88±1.9  33.66±0.81  51.12±3.69  3.59±1.03  42.74±12.76  69.12±5.06  19.9±6.46  34.47±8.35  11.92±4.04  24.35±5.43  37.88±1.42  0.29±0  16.47±5.51  11.55±6.6  9.4±6.1  6.18±2.96  10.42±4.51  24.32±0.83  7.23±4.47  12.12±6.05  2.13±1.45  2.8±1.68  31.25±1.86  27.91±1.66  13.61±9.42  55.11±7.67  13.76±1.19  12.5±7.1  10.04±1.76  12.11±14.71  6.84±4.83  5.87±5.91  26.36±0.6  24.09±4.79  13.07±5.02  21.98±2.05  14.52±7.1  10.42±5.61  15.58±2.28  5.08±1.31  29.17±4.69  24.52±7.25  7.88±4.37  5.3±1.93  27.52±15.97  4.95±0.4  7.95±5.92  18.31±0.63  2.54±0.73  16.48±4.12  24.57±2.11  5.47±0.96  4.17±3.09  17.34±10.21  9.18±4.98  5.87±5.91  11.24±6.31  14.97±1.28  16.48±2.58  4.65±1.94  16.08±2.15  23.3±7.67  29.55±5.82  17.12±8.85  29.55±0  5.91±4.48  7.1±5.53  12.69±1.17  11.92±8.25  1.24±0.6  14.39±9.52  23.8±0.51  10.29±6.19  14.58±7.22  29.36±4.98  7.68±3.55  7.95±2.78  24.9±12.82  7.1±1.06  48.48±2.56  8.62±5.91  6.38±0.66  41.38±36.25  23.84±11.4  9.24±6.87  19.7±1.42  21.41±3.47  5.79±3.84  21.97±2.38  25.68±6.67  2.21±2.72  45.64±5.34  21.8±6.53  4.43±2.47  14.02±1.42  38.08±0.95  9.96±1.66  4.92±2.98  33.33±5.47  19.08±11.69  7.2±5.11  22.97±4.75  9.7±4.39  1.52±0.97  20.83±13.5  29.75±3.11  32.77±8.61  18.02±4.16  14.78±8.65  15.72±7.68  25.87±1.94  9.05±5.31  9.28±0.71  21.22±1.23  4.62±1.53  32.2±6.05  37.6±14.84  3.91±2.66  11.74±6.84  16.45±2.45  1.5±0.88  14.77±0.46  13.31±0.47  2.41±1.86  20.64±2.56  31.4±25.4  7.23±4.22  27.84±5.1  15.6±8.76  6.25±4.66  38.26±6.98  21.51±2.61  19.4±14.55 |

Note: ^a^ E: crude sample extracted with ethyl acetate; M: crude sample from mycelium; W: crude sample from water layer.

**Table S6 Information on genera distribution of actinobacterial strains.**

| Genera | No. of isolates | No. of strains for assay | No. of strains with antimicrobial activity | No. of strains with Immunosuppressive activity | No. of strains with antitumor activity | No. of strains with biosynthetic gene clusters |
| --- | --- | --- | --- | --- | --- | --- |
| *Streptomyces* | 196 | 56 | 28 | 31 | 50 | 56 |
| *Micromonospora* | 46 | 14 | 5 | 7 | 13 | 14 |
| *Curtobacterium* | 24 | 4 | 3 | 3 | 4 | 4 |
| *Actinomadura* | 5 | 2 | 1 | 2 | 1 | 2 |
| *Sinomonas* | 4 | 2 | 1 | 1 | 2 | 1 |
| *Nocardia* | 4 | 3 | 0 | 1 | 3 | 3 |
| *Gordonia* | 3 | 2 | 1 | 2 | 2 | 2 |
| *Rhodococcus* | 2 | 1 | 0 | 1 | 1 | 1 |
| *Microbacterium* | 1 | 1 | 0 | 0 | 1 | 1 |
| *Mycobacterium* | 2 | 2 | 0 | 0 | 2 | 2 |
| Total number | 287 | 87 | 39 | 48 | 79 | 86 |


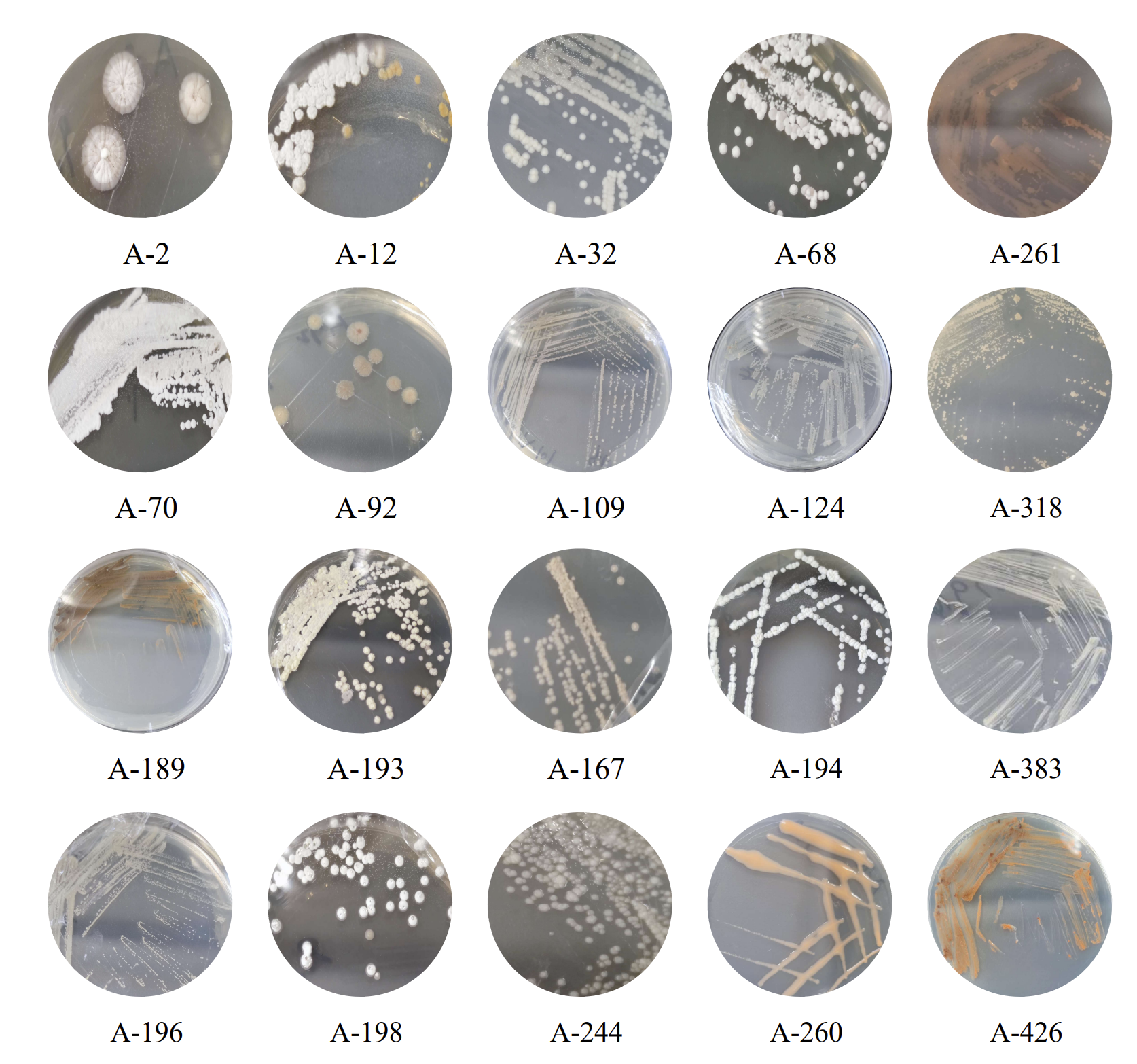


**Figure S1 Colony phenotypes of actinobacterial isolates in this study grown in ISP2 medium.**

| A | B |
| --- | --- |
| 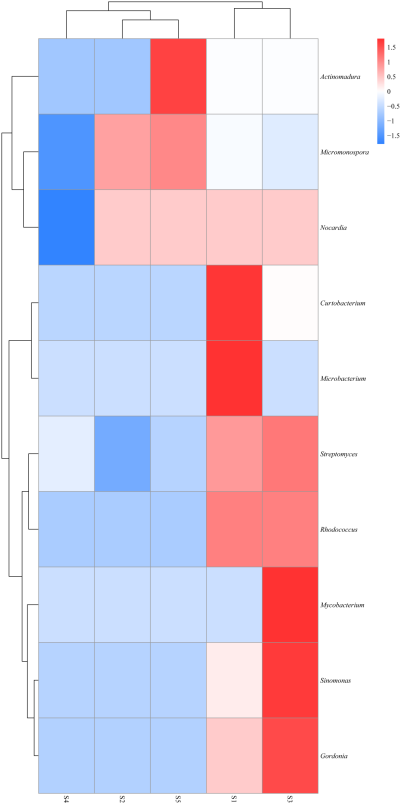 | 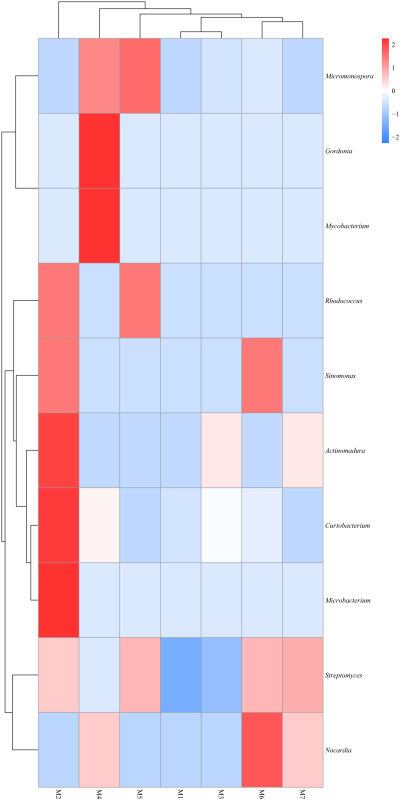 |

**Figure S2 Heat-map of species-based abundance distribution of culturable Actinobacteria from mangrove rhizosphere soils from Hainan island. (A) Species abundance distribution in different sampling sites. (B) Species abundance distribution according to the culture media used for isolation.**

| A | B |
| --- | --- |
| 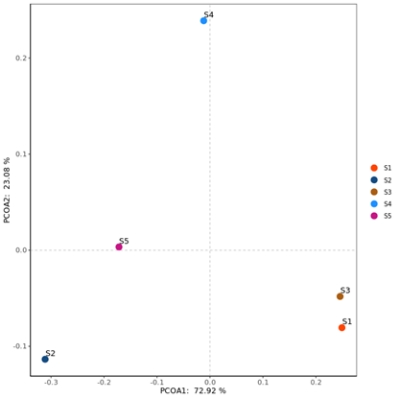 | 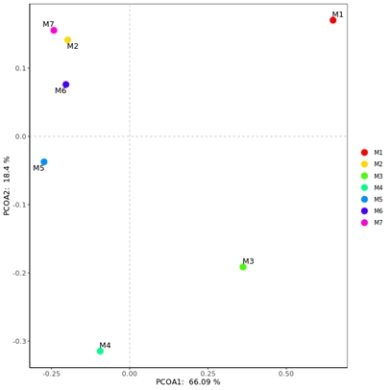 |

**Figure S3 PCoA analyses using bray curtis matrix constrained to (A) sampling sites and (B) culture media used for isolation.**


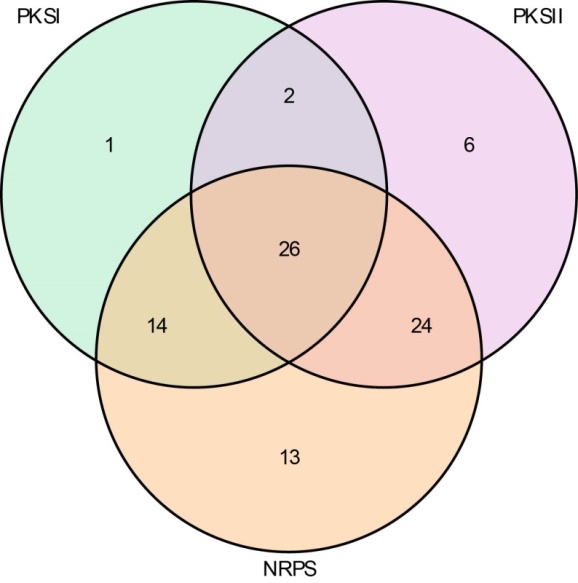


**Figure S4** Venn diagram indicated actinobaterial isolates harboring different types of biosynthetic gene clusters.


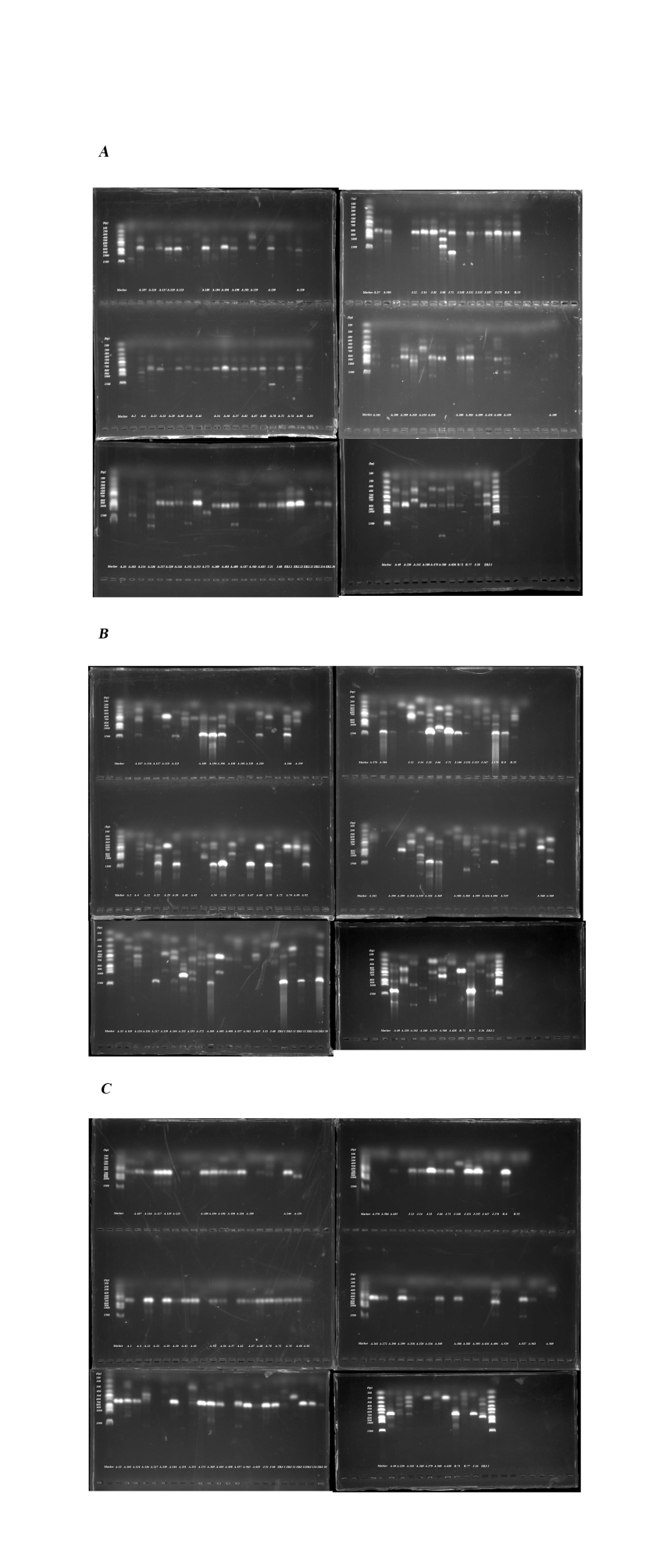


**Figure S5 Biosynthetic gene clusters of culturable Actinobacteria from mangrove soil. (A) NRPS (B) PKSI and (C) PKSII.**
